# Extruded Porous Protein–Lignocellulosic Blends as Fully Bio-Based Alternative to Single-Use Absorbent Plastics

**DOI:** 10.1021/acsapm.5c02445

**Published:** 2025-09-23

**Authors:** Athanasios Latras, Pamela F. M. Pereira, Amparo Jiménez-Quero, Karin Odelius, Mercedes Jiménez-Rosado, Antonio J. Capezza

**Affiliations:** † Department of Fibre and Polymer Technology, 7655 KTH Royal Institute of Technology, Stockholm SE-100 44, Sweden; ‡ Division of Industrial Biotechnology, Department of LIFE Sciences, 11248 Chalmers University of Technology, Gothenburg SE-412 96, Sweden; § Department of Applied Chemistry and Physics, University of Leon, Leon ES-24009, Spain

**Keywords:** porous materials, single-use absorbents, biopolymer blends, biofillers, extrusion

## Abstract

Sustainable technologies have enabled the production of degradable single-use plastics (SUPs) for various applications. However, environmentally friendly, porous disposable absorbents still lack the competitive functionality of synthetic options. In this work, we report the continuous extrusion of fully biopolymer-based porous absorbents derived from integrated proteins and lignocellulosic residues, all sourced from biomass waste. The results show that the saline absorption capacity of the extruded materials increases 1.5 times compared to the reference solely by including oat husk, a lignocellulosic byproduct from the food industry. The absorption was further improved 2 times by including a delignification step on the oat husk and wheat bran, demonstrating the importance of the biomass’s chemistry in increasing the material’s absorption. Here, the addition of 20 wt % of Keratin fibers from food waste increases the material’s absorbency to 6.5 g/g, with the ability to retain 2 g/g of the saline solution in its structure, which is also the highest reported value for extruded protein-based formulations so far. This work advances the development of porous absorbent materials with competitive performance, utilizing industrial methods and upcycling undervalued biomass waste into sustainable consumer products. Introducing porous biopolymer-based materials as alternatives to synthetic counterparts used in the hygiene and sanitary industries ensures the return of safe molecules to nature, paving the way for microplastic-free, single-use, porous absorbents.

## Graphical abstract

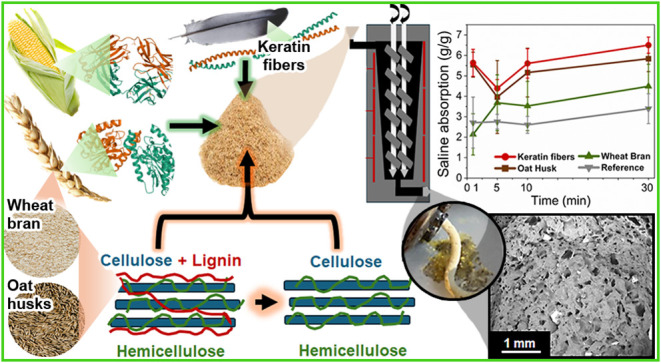

## 1 Introduction

Disposable synthetic absorbents used in everyday hygiene applications pose a threat to the environment due to their fossil-based origin and the large amount of plastic waste generated after their disposal.^1–3^ Simultaneously, the production rates of absorbent hygiene materials are constantly increasing due to higher demand and increased accessibility to these items.^4^ Considering menstrual products and women in their menarche period, approximately 800 million people are end-users of these sanitary pads.^2,5^ Given the number of women who menstruate and the fact that an average person uses 5000 to 15,000 pads or tampons during their lifetime, the generated waste of these synthetic materials can reach approximately 208 million tons globally, which equals about 650 times the weight of the Empire State Building. Furthermore, this waste, which takes hundreds of years to degrade, releases potentially toxic microplastics and carcinogenic acrylic acid monomers.^6–8^

Disposable sanitary products most commonly consist of synthetic superabsorbent polyacrylates (SAP), polyurethane foams (PUR), bleached pulp and polyethylene/polypropylene nonwovens.^9^ In most cases, the postconsumed materials end up in landfills, creating microplastics and polluting both soil and water with substances such as PFAS and phthalates.^10^ Further, the manufacturing process can emit large amounts of carbon dioxide (CO_2_), such as in the case of the U.K., where ca. 13.000 tons of CO_2_ were released only considering the waste management stage.^5,8–12^

Recent reports showcase eco-friendly disposable absorbent alternatives based on agricultural proteins, such as wheat gluten and zein proteins, derived from starch and corn, respectively.^13–15^ These protein blends have been investigated as an alternative to synthetic porous absorbents as promising candidates for single-use absorbents in sanitary pads or diapers.^16,17^ However, the main drawback of extruded protein-based porous absorbents is their limited absorption capacity compared to synthetic materials, and the presence of closed-cell porosity (an unavoidable consequence of the extrusion foaming process). This is unfortunate, as finding adequate alternatives to fossil-based SAPs is urgently needed to permit politicians to take measures for including these types of disposable materials in EU legislation, such as the single-use plastic derivative (SUP).^18^

In this article, we designed fully biobased formulations that incorporate various biofillers upcycled from food waste as an integrated strategy to enhance the liquid absorption functionality of porous extruded materials. The inclusion of biofillers (whether chemically delignified or not) produced extruded porous materials with high open-cell porosity and hydrophilicity. All selected biofillers have an endogenous porous structure, which is advantageous in the context of producing highly porous materials. Oat husk and wheat bran were also selected as strategic raw materials due to their abundance as lignocellulosic residues from the food industry, with 4 and 150 million tons of annual worldwide production, respectively.^19–21^ Likewise, Keratin fibers are a relevant filler to consider as they constitute a significant portion of poultry waste, accounting for approximately 5.9 × 10^6^ metric tons of food waste in 2019.^22^ Remarkably, these materials achieved 29% of the absorption capacity of commercial highly porous alternatives, despite having 72% lower porosity.^23^ The absorption capacity and liquid distribution of the materials were tailored by the presence of oat husk and wheat bran as porous lignocellulosic fillers and Keratin fibers with a rod-like shape, thereby making the matrix more permeable. The extruded materials demonstrated enhanced liquid performance and competitive bioactive properties (here, antioxidant and antimicrobial activity) and are achieved solely through the use of fully renewable resources upcycled from biomass waste. This represents a significant advance toward the development of biobased absorbents for diverse applications, including those currently reliant on synthetic single-use plastics, such as disposable hygiene products.

## 2 Experimental Section

### 2.1 Materials

Wheat gluten protein powder was provided by Lantmännen Reppe AB, Sweden, with a reported 85 wt % protein content (*N* × 6.25), 5.8 wt % starch, 1.2 wt % lipids, 0.9 wt % ash, and 7 wt % water. Zein protein was purchased from Sigma-Aldrich, Sweden (88–96 wt % protein). Oat husk and wheat bran were provided by Lantmännen Reppe AB (Sweden), and Keratin fibers as a byproduct from poultry feathers fermentation were kindly provided by BioExtrax AB (Sweden). Glycerol (ACS ≥98%), sodium bicarbonate (SBC, NaHCO_3_, ACS ≥98%), 5 M sodium hydroxide solution (NaOH, ACS reagent 98%), and hydrogen peroxide (30% w/w in H_2_O, H_2_O_2_) were purchased from Sigma-Aldrich, Sweden. The defibrinated blood was purchased from Håtunalab (Sweden). Milli-Q water (MQw, 18.2 MΩ cm at 25 °C) was used for the delignification and extrusion processes.

### 2.2 Delignification of Wheat Bran and Oat Husk

The delignification of the as-received lignocellulosic biomasses followed the protocol reported by Toquero et al.^24^ based on alkaline hydrogen peroxide treatment (AHP), with some modifications. Ten grams of each biomass was added to a beaker containing 200 mL of MQw (forming a solid:liquid ratio of 1:20) and mixed to remove and wash traces of starch and inorganic components from the biomass. Thereafter, the suspension was washed at 50 °C under stirring (100 rpm) for 1 h (Figure 1a, stage I). After washing, the biomass was filtered and then transferred into prepared solutions of 200 mL of 5% v/v H_2_O_2_ (solid-to-liquid ratio of 1:20, based on the dried biomass weight). The pH was then adjusted to 11.5 using 5 M NaOH, and the reaction was maintained for 1 h at 50 °C under constant stirring (Figure 1a, stage III). After delignification, the biomass was filtered and rinsed with 200 mL of MQ water and dried in an oven at 50 °C for 48 h (Figure 1a, stage V). The liquid residues from the washing and AHP stages were stored in a refrigeration chamber for further characterization. The dried biomass was kept in a desiccator prior to its use. The delignified wheat bran and oat husks were labeled WB-AHP and OH-AHP, respectively. The delignification of WB and OH and the reported results are based on triplicate batches. The entire delignification process is schematically illustrated in Figure 1a. The as-received wheat bran (WB) and oat husk (OH) and the resultant delignified biofillers (WB-AHP and OH-AHP, respectively) were characterized regarding their monosaccharide composition, starch content and phenolic content (see Supporting Information—Biofillers characterization).

**Figure 1:**
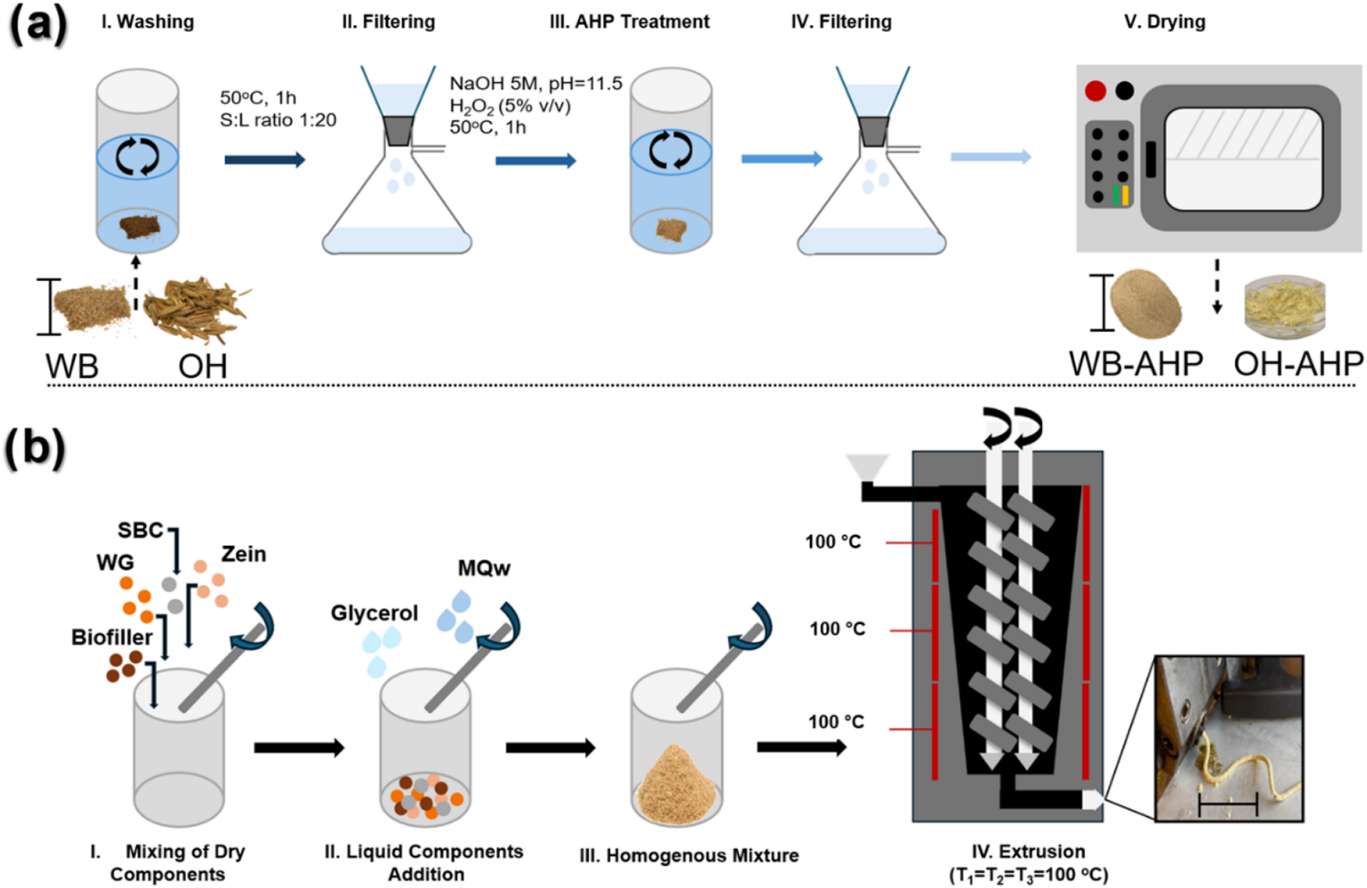
(a) Illustration of the delignification process of the biofillers, wheat bran (WB) and oat husk (OH), and (b) schematic illustration of the formulation’s preparation for extrusion. The scale bar in (a) biomass is 5 cm, and in (b) the extruded filament is 15 cm.

### 2.3 Extrusion of the Biobased Foams

Wheat gluten (WG), Zein (Z), and their blends were mixed with and without SBC, as well as with and without wheat bran (WB), oat husk (OH), WB-AHP, OH-AHP, and Keratin fibers. The labeling for all the extruded formulations prepared is listed in Table 1. In the case of OH, the biomass was first ground into smaller pieces using a grinder with a mesh size of 0.25 mm. All formulations contained 50 wt % glycerol and 5 wt % MQw (based on the protein content), while the protein blend ratio between WG/Z was 25/75. For example, to prepare 100 g of the extruded reference foams, 25 g of WG, 75 g of Z, and 5 g of SBC (solid reagents) were physically mixed, as shown in Figure 1b, stage I. After mixing all the solids, 5 g of MQw and 50 g of glycerol were manually mixed rigorously until a fine mixture was formed (Figure 1b, stage II). For the formulations containing biofillers, 5 wt % of each biomass was added to the formulation described above (independently of whether they were delignified or used as-received). The selected ratios of glycerol, MQw, WG/Z, and SBC used in this study are based on previous research.^16^ The mixture was then manually and gradually added to the hopper of a corotating double screw mini extruder (DSM Xplore 5 cm^3^, The Netherlands) (Figure[Fig fig1]b stage IV). The *L*/*D* ratio was 8, the compression ratio was 3.3, and the die diameter was 2.8 mm. The screw speed was 60 rpm, and all heating zones of the mini extruder were set at 100 °C. All samples were kept in a desiccator for at least 48 h before any characterization.

**Table 1:** Composition of the Extruded Formulations Herein and Their Respective Labeling^a^

formulation	Zein	WG	SBC^b^ (wt %)	Keratin^b^ (wt %)	WB^b^ (wt %)	WB-AHP^b^(wt %)	OH^b^ (wt %)	OH-AHP^b^(wt %)
WG		100		5:10:20	5	5	5	5:10:NE
WG/SBC		100	5	5:10:20	5	5	5	5:NE
Z	100			5:10:20	5	5	5	5:10:20
Z/SBC	100		5	5:10:20	5	5	5	5:10:20
WG/Z	75	25		5:10:20	5	5	5	5:10:20
WG/Z/SBC	75	25	5	5:10:20	5	5	5	5:10:20

a Note: NE means Not Extrudable. All formulations contain 50 g of glycerol and 5 g of MQw per 100 g of protein. Each formulation contains only one biofiller: Keratin, wheat bran (WB), oat husk (OH), and delignified wheat bran or oat husk (WB-AHP and OH-AHP), as specified by composition.

b wt % per 100 g of protein.

### 2.4 Physical Characteristics of the Samples

The microstructure of the as-received and AHP-delignified biomasses, Keratin fibers, and extruded samples was evaluated with a tabletop scanning electron microscope (TM-1000 Hitachi, Japan). The sample preparation for the extruded materials involved immersing the extrudates in liquid nitrogen for 10–20 s and then cryo-fracturing to minimize plastic deformation. The sample’s cross-section and surface were placed on carbon tape for the characterization. The pore and particle size distributions for the extrudates and biofillers, respectively, were defined by measuring the pore sizes and longest dimension of at least 50 pores and particles, using the software ImageJ.

The apparent density of the extruded samples was calculated by a gravimetric method. Each sample was assumed to have a cylindrical shape and the density ρ was reported as kg/m^3^. The reported densities correspond to the average of triplicate measurements from each formulation.

### 2.5 Fourier-Transform Infrared Spectroscopy (FT-IR)

FT-IR analysis was performed to elucidate the characteristic bonds of the proteins and the biofillers (before and after their delignification). The FT-IR spectra were obtained with a PerkinElmer Spectrum 100. The scan resolution was 4.0 cm^–1^ with a scanning step of 1.0 cm^–1^. The profile was obtained from 16 consecutive scans between 4000 and 600 cm^–1^.

### 2.6 Liquid Swelling Performance

Initially, a visual absorbance test (VAT) was conducted for all raw components and the delignified biomasses, i.e., WG, Zein, OH, WB, Keratin, WB-AHP, and OH-AHP. 1–2 mg of the material was placed on a Petri dish, and 100 μL of saline solution (0.9 wt % NaCl) and defibrinated sheep blood were gradually added until the saturation point was reached (i.e., when the liquid visually leaked from the material). The VAT was performed in triplicate, and the average values were calculated as the amount of the liquid absorbed by the materials (g) divided by the dry weight of the material (g).

The free swelling capacity (FSC) was estimated following the nonwoven standard procedures (NWSP 240.0.R2). 200 mg of the dry materials (*W*_d_) were placed into a nonwoven PP/PE plastic tea bag (*W*_b_ dry bag weight) and then immersed in saline solution (0.9 wt % NaCl) for 1, 5, 10, and 30 min. After the respective swelling time, the material was kept for 10 s out of the solution, then gently placed on tissue paper for 10 s, and finally weighed (*W*_w_). The same procedure was repeated for empty bags to correct the FSC values, taking into account the intrinsic capillary absorption of the wet bag (CF). The FSC values were calculated based on triplicate measurements, following eq 1.

1 $$FSC(g/g)=\frac{W_{w}-\left(W_{b}\times CF\right)-W_{d}}{W_{d}}$$

The 30 min FSC materials were centrifuged at 1200 rpm for 3 min to determine their centrifuge retention capacity (CRC) according to the NWSP 240.0.R2 standard. After 30 min of swelling, the CRC value was determined as the ratio of the weight after centrifugation (*W*_crc_) to the weight of the dry material (*W*_d_), as shown in eq 2. All samples for the CRC values were carried out in triplicate.

2 $$CRC\left(g/g\right)=\frac{W_{crc}-W_{d}}{W_{d}}$$

### 2.7 Thermal and Mechanical Properties

The thermal properties of the formulations used herein (before extrusion) were evaluated using a TGA/DSC 1 (Mettler Toledo). Samples of 5 mg were heated between 25 and 800 °C in a nitrogen atmosphere at a heating rate of 10 °C/min.

Rheological tests were performed to evaluate the viscoelastic behavior of the samples in shear mode. To have fine and uniform surfaces between the material and the plates of the rheometer, the mixtures were prepared as described in Section 2.3 and then transferred into a 10 mm thick mold with 12 cavities of 25 mm diameter (see Figure S1). Poly­(tetrafluoroethylene) (PTFE) sheets were placed on both sides (top and bottom) of the mold to prevent the material from sticking. Then, the mold was placed between two metallic plates to distribute the pressure homogeneously. Approximately 2 g of each formulation was placed in each cavity, and then pressure was applied, 100 kN for 2 min at 20 °C.

Rheological measurements were performed using a TA Instruments Discovery HR-2 rheometer with a 25 mm diameter parallel plate and a Peltier plate for temperature control. The gap was fixed at 1000 μm. First, strain sweep tests were performed at 1.0 Hz, 20 °C, and a strain range of 0.002–2% to evaluate the linear viscoelastic range (interval in which the elastic (*E*′) and viscous (*E*″) moduli remain unchanged). Frequency sweep tests were also conducted at a constant strain (within the linear viscoelastic range) at 20 °C and a frequency range of 0.2–20 Hz. Finally, temperature ramps were performed from 25 to 150 °C at a heating rate of 10 °C/min, 1.0 Hz and a strain within the linear viscoelastic range. The Trios v.4.21 software was utilized for data acquisition. All samples were run in duplicates.

A tensile test was performed on the extruded materials using an Instron 5944 (single-column) universal testing machine with a 500 N load cell. The extension rate was 10 mm/min. The samples that provided the highest absorbance results were chosen for the tensile test. For each sample, five specimens from the extruded filaments (ca. 10 cm long) were tested. The data of elastic modulus (*E*) were obtained from the slope of the linear region, while the tensile stress (σ_b_) and elongation at break (ε_b_) were measured at the highest value of stress and the strain at the last point before break. All specimens were conditioned at 50% RH and 23 °C for 48 h prior to the test.

### 2.8 Bioactivity Properties

The bioactivity of the developed materials was evaluated in terms of antioxidant activity and antimicrobial properties, and the reported results are based on triplicates. The antioxidant activity was measured as the scavenging activity against the radical DPPH (1,1-diphenyl-2-picrylhydrazyl) according to Brand-Williams et al.,^25^ with a few modifications. The assay was performed with the different protein-based materials over three cycles of oxidation to determine whether the antioxidant property performance could be maintained over time. For this, 2 mL of a 0.1 mM methanolic solution of DPPH was used, and the results were monitored through three DPPH addition cycles at 15 min intervals. The results were promptly determined at 517 nm, using a microplate reader FLUOstar (BMG Labtech, Germany).

The disk diffusion assay was used to complementarily evaluate the antimicrobial activity of the developed materials, aiming to determine whether the potential properties are associated with a diffusive material or result from direct contact with the surface. For this, *Escherichia coli* (CCUG 10979), a Gram-negative bacterium, and *Bacillus cereus* (CCUG 7414) and *Staphylococcus epidermidis* (CCUG 39508T), Gram-positive representatives, were studied. The bacteria strains were cultured in Lysogeny Broth (LB) media. The cell density concentration was adjusted to a McFarland scale of 0.5 using a spectrophotometer. For this, 100 μL of the inoculated media was spread on the surface of solidified Mueller-Hinton agar plates. Subsequently, the previously cut triplicate of the samples was placed on the dried surface of the plates. The assessment of antimicrobial activity involved measuring the diameter of the antibacterial inhibition zone and observing bacterial growth on the contact surfaces of the material after a 24-h incubation period at 37 °C.

### 2.9 Statistical Analysis

Statistical analyses were performed using the least significant difference (LSD) in Fisher’s procedure to evaluate the significance of the measurements (*p* < 0.05, 95% confidence level). These tests were analyzed using Statgraphics 18 software. The results were represented as mean values with standard deviations, indicating significant differences between superscript letters (*P* ± SD*^x^*). At least triplicate samples were used in the analysis.

## 3 Results and Discussion

### 3.1 Physicochemical Role of the Biofiller as Absorbents

The peroxide alkaline delignification of wheat bran (WB) and oat husk (OH) yielded 22% and 51% of delignified biomass, respectively (based on their initial mass, see Figure S2). The high mass loss is attributed to the removal of starch traces and water-soluble components, such as sugars and salts.^26^

The delignification, combined with a washing process, was responsible for removing approximately 51% of the lignin content in WB and OH (based on comparing the lignin content of the washed and postdelignified wheat bran and oat husk). The washing step was particularly effective for WB, having an initial starch content of 186 ± 30 mg g^–1^, in contrast to OH, which had a starch content of 2.38 ± 0.91 mg g^–1^ (see Table 2). Starch removal was confirmed by the increased in the FTIR peak at 1000 cm^–1^ in the washing solution (see Figure S3). Furthermore, Figure 2a1,b1 (and Figure S4) show an increase in porosity observed in the microstructure of the WB and OH, resulting after the removal of lignin and hemicellulose, which aligns with previous studies.^24,27^ To enhance process circularity, the washing solution is suggested to be tested as a nutrient-rich fertilizer, while black liquor could be repurposed for further delignification or antioxidant extraction.^27,28^

**Figure 2:**
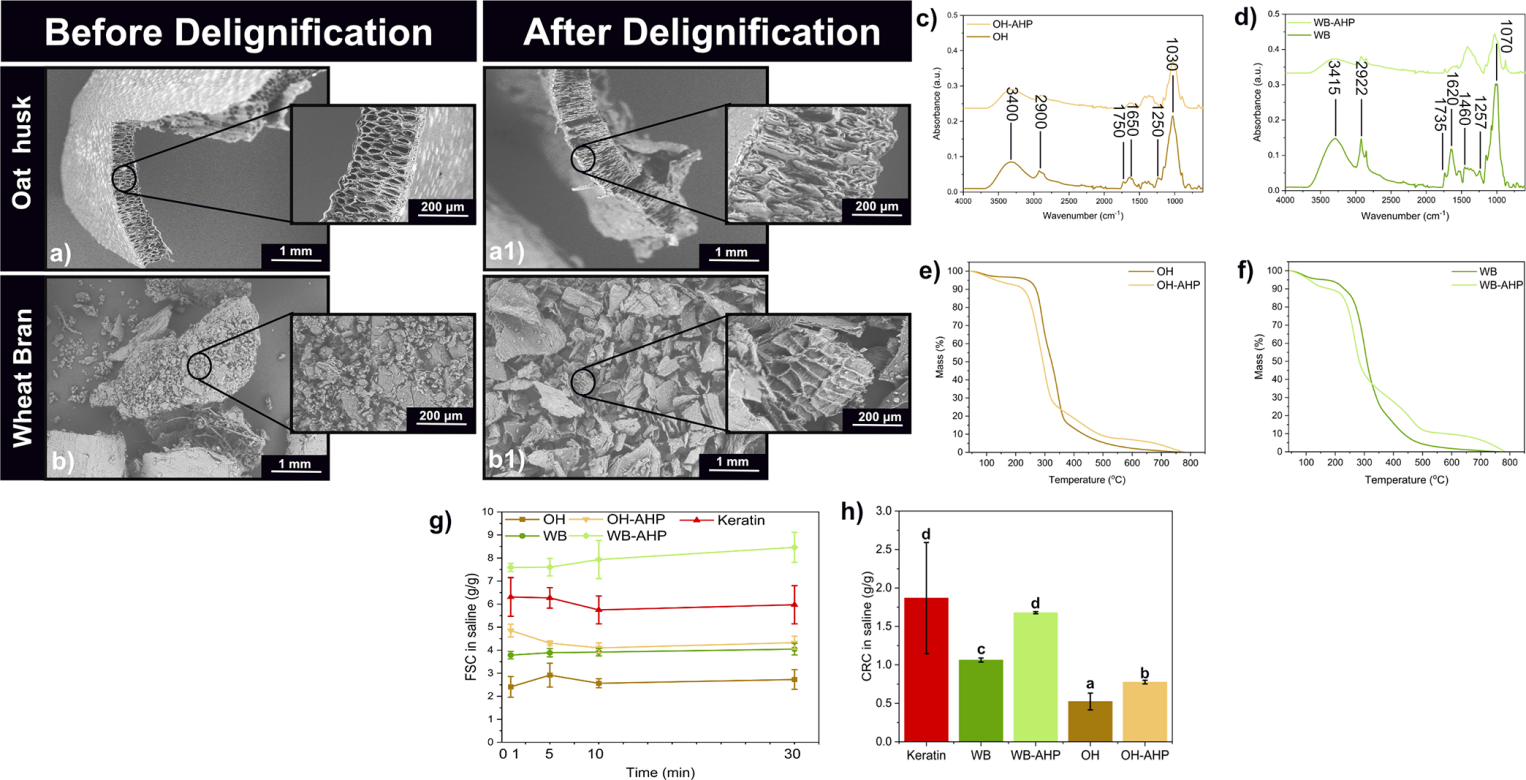
(a, b) Microstructure of the as-received wheat bran (WB) and oat husk (OH) and (a1, b1) after delignification. (c, d) FT-IR spectrum of as-received and delignified OH (OH-AHP) and WB (WB-AHP), and (e, f) their TGA profiles. (g) Free swelling capacity (FSC) in saline (0.9 wt % NaCl) and (h) centrifuge retention capacity (CRC) of Keratin fibers (Keratin) and oat husk (OH), and wheat bran (WB), before and after delignification (OH-AHP and WB-AHP). Note: Different letters (a–d) in Figure 2h mean that the values are significantly different (*P* < 0.05).

**Table 2:** Characterization of Wheat Bran (WB) and Oat Husk (OH) Used as Biofillers in the Protein-Based Foams^a^,^b^

composition (mg/g)	WB	WB-W	WB-AHP	OH	OH-W	OH-AHP
total carbohydrates	650.45 ± 156.03	-	861.93 ± 149.85	570.60 ± 82.68	-	679.89 ± 24.39
cellulose	257.13 ± 23.56	-	46.27 ± 10.05	245.61 ± 11.31	-	199.80 ± 4.15
starch	186.15 ± 30.12	-	3.47 ± 0.46	2.38 ± 0.91	-	n.d.
phenolic compounds	39.79 ± 12.37	-	1.99 ± 0.14	175.60 ± 34.45	-	17.25 ± 1.07
total lignin content	290.86 ± 7.94	368.29 ± 23.14	178.81 ± 4.07	317.60 ± 6.62	322.63 ± 10.71	155.49 ± 12.21

a The labels correspond to the biomasses as-received (WB and OH), washed (WB-W and OH-W) and after delignification (WB-AHP and OH-AHP).

b Note: n.d. represents a not detectable result.

Figure 2c,d shows the FTIR spectra of as-received and delignified WB and OH. In the as-received biofillers, the broad absorption bands at 3415 cm^–1^ and 3400 cm^–1^ correspond to hydroxyl groups in cellulose, hemicellulose, and lignin.^20^ Peaks at 2922 and 2900 cm^–1^ relate to CH_2_ stretching in lignin and cellulose.^29^ Delignified WB (WB-AHP) and OH (OH-AHP) show decreased absorption in the 1000–1750 cm^–1^ region, particularly C=O and aromatic stretching vibrations, indicating lignin removal.^21,28,30^

Delignification affected the thermal stability of WB and OH (Figure 2e,f). Initial weight loss at 100 °C (90–92%) corresponds to moisture evaporation, followed by the decomposition of hemicellulose, cellulose, and lignin. Hemicellulose starts decomposing slightly earlier than cellulose, which decomposes between 300–400 °C, while lignin degrades more gradually over a wider range due to its aromatic structure.^31–33^ Delignified biofillers decompose at 20–30 °C earlier, attributed to the removal of lignin.^34^ The increased in inorganic residue observed in WB-AHP and OH-AHP can be attributed to the delignification process itself. Since the treatment primarily removes organic constituents (cellulose, hemicellulose, and lignin), the overall sample mass decreases, while the inorganic components remain unaffected. Consequently, their relative proportion in total mass increases (see Figure 2e,f). Here, typical inorganic components reported in OH and WB are silica (SO_2_, absorbed by the plant) and phosphorus/potassium phosphates (essential minerals found in wheat bran), respectively.^35,36^

The FTIR spectra of the as-received Keratin fibers (Figure S5a) show the absorption bands associated with O–H and C–H stretching, along with the characteristic amide I, II, and III groups of proteins.^37^ The TGA profile indicates moisture loss at 100 °C, degradation near 300 °C, and the formation of an inorganic residue above 350 °C (Figure S5b). Surface cleft lines on the fiber’s surface suggest enhanced interfacial bonding potential with the protein blend^38,39^ (see Figure S5c). Overall, the thermal stability of the biofillers tested herein (i.e., OH, WB, and Keratin fibers), whether delignified or as-received, falls within the extrusion processing window, allowing for further processing.

The absorbency of the biofillers (without the protein matrix) was tested in saline and defibrinated sheep blood (see Figures 2g,h and S6a,b). Keratin fibers showed the highest VAT (Visual Absorbance Test) (11–12 g/g) and FSC in saline (6 g/g), followed by WB (4 g/g) and OH (2.5 g/g). A similar trend was observed for CRC, with Keratin (1.8 g/g) outperforming WB (1 g/g) and OH (0.5 g/g) (see Figure 2h). The delignification significantly enhanced WB and OH absorbency, doubling that of the nontreated WB’s FSC (from 4 to 8 g/g) and increasing OH’s FSC by 2 g/g (Figure 2g,h). These results highlight the potential of delignified biomass and upcycled Keratin to create competitive, renewable absorbent layers, addressing a key limitation of sustainable porous materials.^13,17^ The FSC in saline reached 36% of that of polyurethane foam from a commercial menstrual pad, the closest reported value for replacing synthetic absorbents with food waste-derived alternatives.^23^

### 3.2 Influence of the Biofiller on the Absorption of the Extruded Materials

The FTIR spectrum of Wheat Gluten (WG) and Zein (Z) confirms the presence of characteristic Amide I and II absorption bands^40,41^ (Figure S7a)· Simultaneously, the TGA of both WG and Z shows that these undergo a major weight loss between 250 and 300 °C, reaching a 50% mass loss at 325 °C (see Figure S7b,c), which is in agreement with previous results.^42,43^ This indicated that the as-received proteins are suitable for thermal processing at the selected temperature (Figure 1b, step 4).

Extruding the protein blend with the as-received biofillers at a fixed content (i.e., 5 wt %) resulted in a microstructure similar to of the extruded reference, with no evidence of biofiller aggregation regardless of the type of biofiller (OH, WB, Keratin, see Figure S8). Moreover, the addition of the OH, WB, and Keratin to the WG did not considerably vary the screw force of the reference extruded gluten and only partially increased the force on the Z systems (see Table S1). However, the presence of the biofiller enhanced the swelling performance of the extruded materials in both saline and blood (Figure S9), and noticeably increased the retention capacity in saline, compared to the reference by 1.3 times (Figure S9c2). Here, it is observed that the presence of Z in the formulations allows for a higher retention of glycerol after swelling compared to only using WG (Figure S6c), which is a typical phenomenon observed in previous reports.^17^ The results suggest that Z not only influences the microstructure of the material but also the retention of the plasticizer. Additionally, the density of the samples did not change significantly when the as-received biofillers were included (see Table S2). Hence, it is shown that the inclusion of the biofillers plays a key role in influencing the swelling and retention performances of the protein-based porous materials (Figure S9).

The homogeneous structure of the extruded materials containing well-distributed WB and OH embedded in the protein network can be ascribed to the lignocellulosic biomass providing hydroxyl groups, which can potentially facilitate hydrogen bonding and electrostatic interactions with the polar side chains of the proteins.^44^ On the other hand, Keratin fibers, due to their proteinaceous nature, could engage in protein–protein interactions, including hydrogen bonding, or even potentially disulfide exchange with wheat gluten or between their common tyrosine residues.^45^

Introducing 5 wt % of the delignified WB and OH (i.e., WB-AHP and OH-AHP) resulted in extruded products having 1.25 times higher free swelling values compared to those including as-received biofillers, as shown in Figure 3a–c. The microstructure of the WG-based formulations does not show a porous structure even with the addition of SBC, as a foaming agent (Figure 3d,d1). On the other hand, the formulations with Z (Z/SBC-WB-AHP and WG/Z/SBC–OH-AHP) developed highly porous structures with the addition of SBC as a foaming agent (Figure 3e,e1 and f,f1), which is reported to impact the liquid swelling performance of extruded materials positively.^46^ The influence of SBC can also be observed in the density and expansion values of the extruded formulations (Table S3). The lowest density values were obtained by the blends containing SBC, i.e., WG/Z/SBC-WB-AHP, WG/Z/SBC-WB–OH-AHP, and WG/SBC-WB-AHP, with densities of 608, 574, and 666 kg/m^3^, respectively. These formulations also exhibit the highest expansion ratios among all materials (see Table S3).

**Figure 3:**
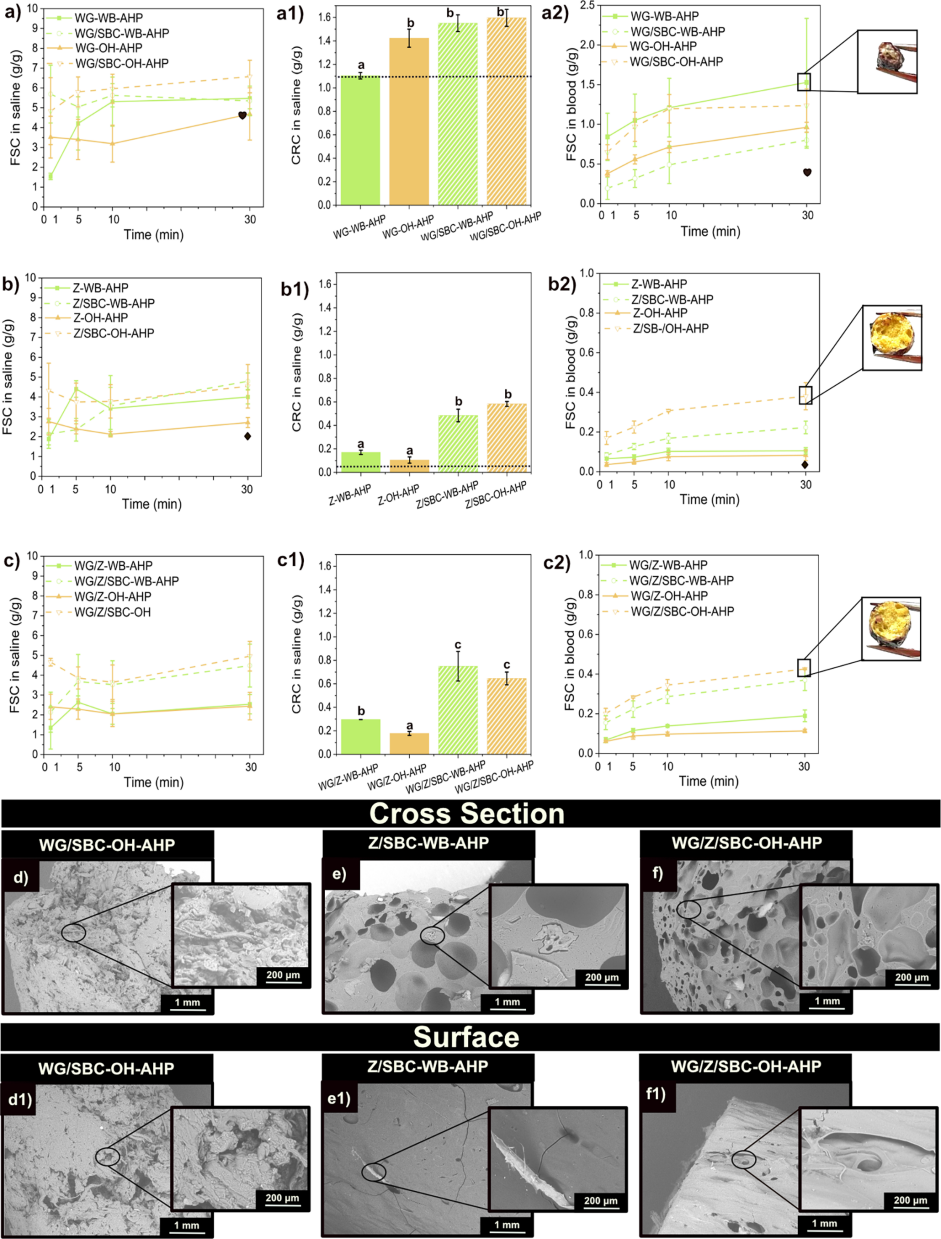
(a–c) Free swelling capacity (FSC) (a1–c1) and centrifuge retention capacity (CRC) in saline solution and (a2–c2) Free swelling capacity (FSC) in defibrinated sheep blood of the samples WG, WG/SBC, Z, Z/SBC, WG/Z, and WG/SBC/SBC with WB-AHP and OH-AHP. Note: The symbols “heart” and “diamond” represent the FSC values of WG and Z at 30 min, respectively, and the dotted line shows the CRC value of WG and Z, respectively. Different letters in Figure 3a1–c1 mean that the values are significantly different (*P* < 0.05). (d–f and d1–f1) Microstructure of the protein formulations with the delignified wheat bran (WB-AHP) and oat husk (OH-AHP) biofiller with SBC. The samples displayed here are those that resulted in the highest free swelling performance.

When WG/SBC was combined with OH-AHP, the liquid uptake increased to 1 g/g within 30 min (see Figure 3a). Figure 3a shows that the addition of WB-AHP did not affect the free swelling behavior of the WG-based material. On the other hand, including WB-AHP in the Z-based samples (Z/SBC-WB-AHP) increases saline uptake, reaching 2 g/g within 30 min (Figure 3b). In all formulations, SBC played a critical role since the formation of pores allows the liquid to penetrate the structure more easily. The formulations, both with WB-AHP or OH-AHP (i.e., WG/SBC, Z/SBC, and WG/Z/SBC), exhibit a higher retention capacity compared to their reference formulations (i.e., without SBC). The delignification had a significant effect on increasing FSC of the samples in saline, while showing similar density values and microstructure with those of the as-received OH and WB (i.e., WG/Z/SBC–OH 4.5 g/g and ρ = 552 kg/m^3^ and WG/Z/SBC–OH/AHP 5 g/g and ρ = 574 kg/m^3^).

The free swelling in blood and retention in saline (CRC) of the extruded protein foams containing the delignified biomass are displayed in Figure 3a2–c2. For the Z and WG/Z-based formulations, the inclusion of delignified oat husk (OH-AHP) resulted in the highest FSC/CRC, as seen in both Z/SBC–OH-AHP and WG/Z/SBC–OH-AHP, which have a value of 0.4 g/g (Figure 3b2,c2). The lower FSC values in blood compared to saline can be explained by the fact that blood consists of a mixture of polar and nonpolar molecules. Furthermore, as seen in Figure 3d1–f1, the surface of the extrudates is rather solid, which can impair their swelling by blocking the blood from accessing the inner part of the filaments (see insets in Figure 3b2,c2). Forthcoming work can focus on increasing the pore size on the material’s surface to achieve higher swelling values in blood. Nonetheless, the addition of WB-AHP and OH-AHP in the protein matrix increased the porosity within the cell walls of the materials, as seen in Figure 3d–f.

Overall, the extruded materials show a similar porous structure regardless of whether the biomass is delignified or used as received (Figures 3 and S8, respectively). Therefore, the results suggest that the increase in liquid absorption for the systems containing WB-AHP and OH-AHP could be a consequence of the decrease in lignin content, resulting in higher polar liquid affinity. Here, the delignification has been reported to expose more of the hydroxyl groups owned by cellulose or hemicellulose. As a result, more hydrogen bonding interactions could be formed, which can impact both the protein/biofiller interaction and its liquid uptake behavior^32,35^ (see Figure 3a–c). Subsequent work should focus on identifying specific interactions between these biofillers and the protein matrix, for example, using FT-IR, Circular Dichroism (CD), and solid-state NMR, toward proposing a mechanistic behavior for the increase in liquid swelling beyond the changes in the porosity of the materials.

### 3.3 Influence of the Biofiller Content on the Material’s Absorption

To investigate whether the biofiller content plays a role in the absorbance properties of the extruded materials, the OH-AHP combined with WG/Z/SBC was selected due to this system showing the highest FSC and CRC in saline among the samples tested. The least performing sample (Keratin combined with WG/Z/SBC) was also included for comparison purposes (see Figure 4). The extrudates having the highest FSC values were those containing OH-AHP at 10 wt %. This can also be explained by the high porosity of the samples with OH-AHP (Figures 4d–f and S10), compared to Keratin-based samples (Figure S11). The sample WG/SBC–OH-AHP-10 was not extrudable (WG containing SBC and 10 wt % of OH-AHP), while without SBC (WG–OH-AHP-10), it presented a tightly packed microstructure (Figure 4d1). The dense microstructure is a possible consequence of the high content of OH-AHP and high elasticity of WG protein, which could collapse the porous structure and make the whole material more compact (Figure 4d). The Z/SBC–OH-AHP-10 (10 wt % of OH-AHP) showed higher porosity with a greater average pore size (223 μm, Table S4) compared to when 5 wt % OH-AHP was used (Figure 4e). Overall, increasing the biofiller content to 10 wt % led to higher FSC values, while the physical properties, such as density, remained nearly unchanged (Table S4).

**Figure 4:**
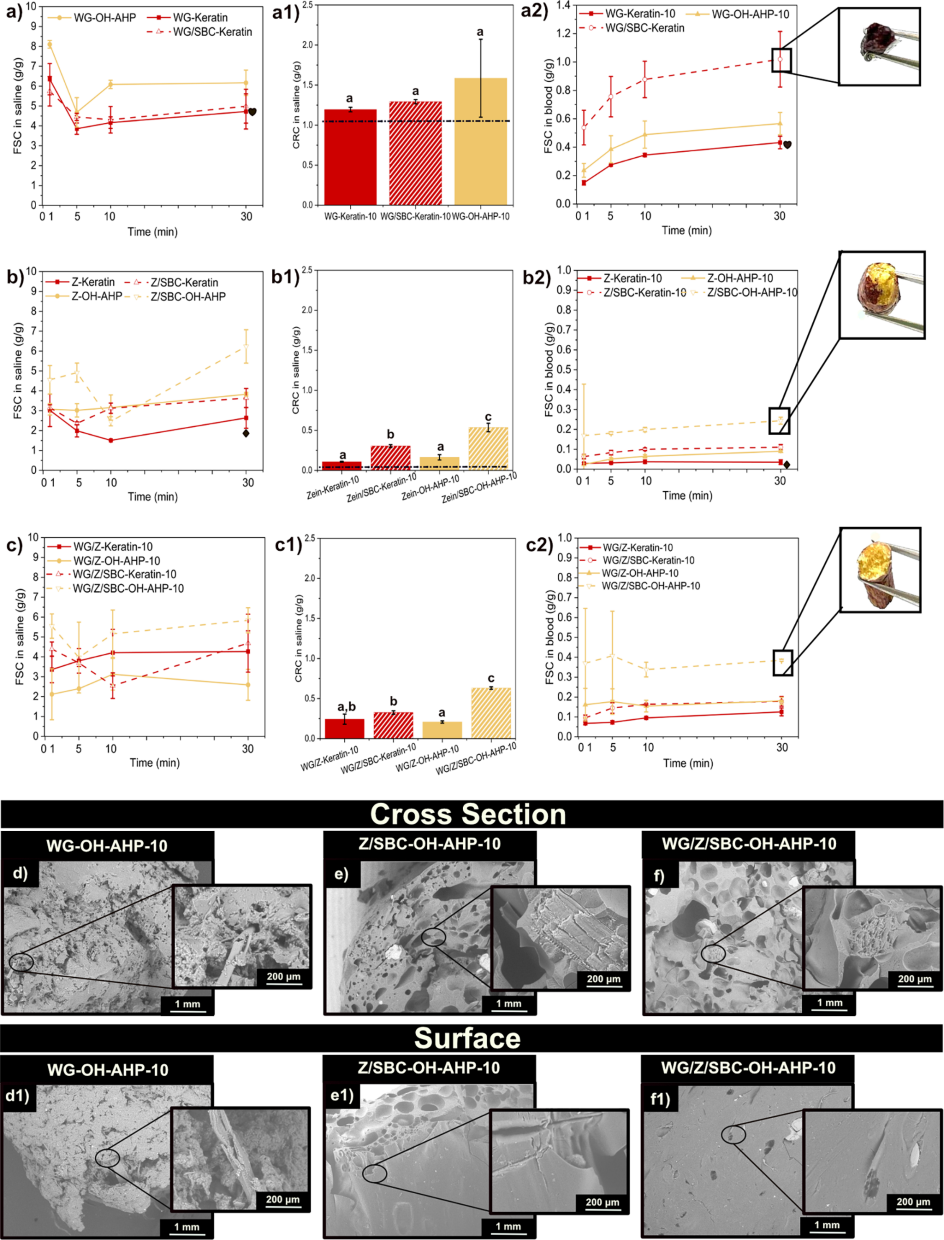
(a–c) Free swelling capacity (FSC) in saline, (a1–c1) centrifuge retention capacity (CRC) in saline, (a2–c2) and FSC in blood for the samples WG and WG/SBC, Z, Z/SBC, WG/Z and WG/Z/SBC with 10 wt % of Keratin and delignified oat husk (OH-AHP). Note: The symbols “heart” and “diamond” represent the FSC values of WG and Z at 30 min, respectively, and the dotted line shows the CRC value of WG and Z, respectively. Different letters in Figure 4a1–c1 mean that the values are significantly different (*P* < 0.05). (d–f and d1–f1) Microstructure of wheat gluten (WG), Zein (Z), and protein blends with delignified oat husk (OH-AHP) at a 10 wt % content of the biofiller with SBC. Only the recipes exhibiting the highest free swelling performance are shown.

The retention capacity of the samples did not change significantly between the two different contents of the biofillers. The free swelling in blood for the OH-AHP samples yields similar results to those with a 5 wt % content (Figure 4 a2–c2). However, increasing the Keratin fiber content to 10 wt % with WG and SBC resulted in the highest FSC in the blood (1 g/g, Figure 4a2), being the only recipe that showed efficient penetration of the blood inside the cross-section. The results demonstrate the role of Keratin in facilitating blood penetration into the porous structure, which is a significant drawback of previous porous protein absorbents used in hygiene products.^16^

For samples containing 20 wt % of the biofiller, the highest swelling results were dominantly obtained when the samples were combined with Keratin (see Figure 5). Furthermore, at this high biofiller content, the WG-based formulations with OH-AHP were not extrudable. On the contrary, Z/SBC–OH-AHP-20 is extrudable and shows high porosity and large pores (Figure 5e), while its density is still at the same level as it was with 5 wt % of OH-AHP (Table S5). The addition of 20 wt % Keratin in the protein blend resulted in a highly porous structure containing well-distributed Keratin fibers around the material cell walls (Figure 5f) and a slight increase in its density (850 kg/m^3^, Table S5). Figure S13 shows that increasing the content of the biofiller does not lead to a significant difference in the pore size distribution. However, the presence of SBC and the specific protein used (WG or Z) changes the pore size distribution (Figure S13).^13^

**Figure 5:**
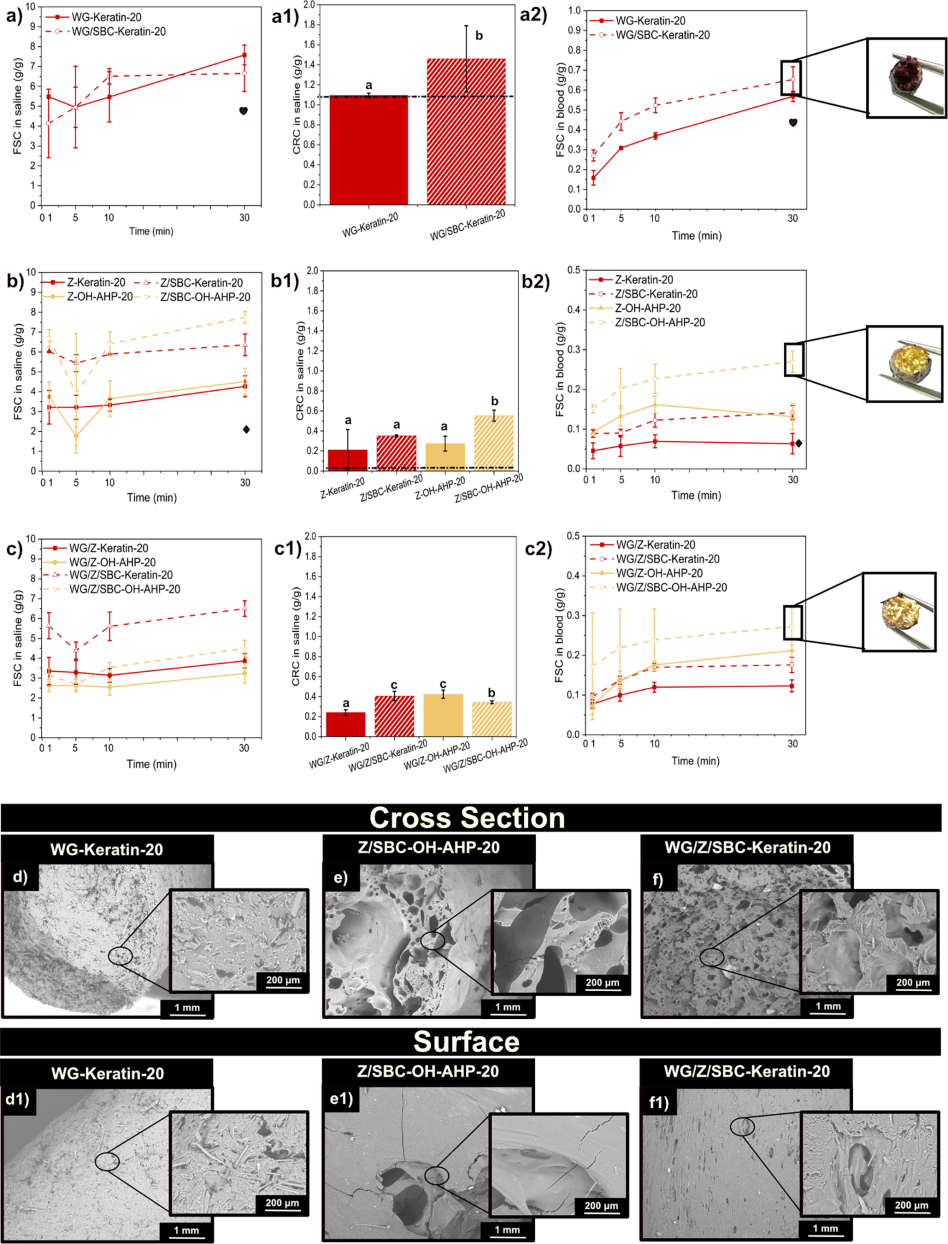
(a–c) Free swelling capacity (FSC) in saline, (a1–c1) centrifuge retention capacity (CRC) in saline, and (a2–c2) FSC in blood for WG and WG/SBC, Z and Z/SBC, WG/Z and WG/Z/SBC samples containing 20 wt % of Keratin and delignified oat husk (OH-AHP). Note: The symbols “heart” and “diamond” represent the FSC values of WG and Z at 30 min, respectively, and the intermittent line shows the CRC value of WG and Z, respectively. Different letters in Figure 5a1–c1 mean that the values are significantly different (*P* < 0.05). (d–f and d1–f1) Microstructure of the protein formulations with Keratin and delignified oat husk (OH-AHP) at 20 wt % biofiller. Only the recipes exhibiting the highest free swelling performance are shown.

The saline swelling of WG samples containing Keratin (with/without SBC) increased to 6.5 and 7 g/g, respectively, while the retention capacity reached 1.4 g/g with SBC (Figure 5a1–c1). For the Z-based formulations, Z–OH-AHP and Z-Keratin portrayed a swelling of 4 g/g, while in the presence of SBC, it reached 7.5 and 6 g/g, respectively (Figure 5b). This significant FSC value in Z-Keratin-20, compared to Z/SBC-Keratin, can also be explained by the development of a porous surface even without SBC (see Figure S12a). Moreover, the retention ability of the samples was higher when combined with OH-AHP (Figure 5b1). Lastly, the protein blend achieved the highest uptake reported, with 20 wt % Keratin reaching 6.5 g/g (Figure 5c,c1).

Furthermore, the moisture content of the extruded materials was gravimetrically evaluated by drying for 24 h postextrusion and after storage for 6 months at room temperature (30–50% RH), resulting in less than 1% and approximately 9% moisture content, respectively. Similarly, no structural changes or microbial activity were observed in these stored samples after visual inspection, supporting their storage stability. Future work should, however, address correlations between microstructure and performance stability.

Here, the addition of 20 wt % Keratin (WG/Z/SBC-Keratin-20) resulted in an approximately 40% increase in saline FSC compared to previously reported gluten-based absorbent foams produced by oven expansion for sanitary applications. At the same time, the maximum FSC reported herein is one-third of the absorption capacity of the absorbent PUR foam layer extracted from a commercial sanitary pad, underscoring its practical relevance relative to synthetic benchmark materials.^13,23^ Furthermore, the WG/Z/SBC-Keratin-20 sample exhibited increased swelling after prolonged exposure to saline solution and sheep blood, and also after being dried postswelling and reswelled (see Figure S15). Although this work focuses on single-use absorbents, these findings open opportunities for further exploration in applications where repeated swelling is relevant, such as agriculture and vertical farming.^47^

Despite the absorption capacity being 1/3 of that of synthetic foam references, a key performance indicator of these extruded materials compared to commercial foams is their demonstrated ability to undergo both hydrolytic and soil biodegradation within weeks, thereby eliminating the risk of microplastic generation.^48^ The incorporation of biofillers in the formulations is not expected to compromise this inherent degradability. Nonetheless, upcoming studies could systematically evaluate the impact of these biofillers on the foams’ biodegradation kinetics, particularly for applications where porous absorbents with controlled biodegradation rates may play a key role, such as in agriculture.

It is worth pointing out the challenges of developing materials from biomass, especially when moving toward pilot-scale testing.^49^ Relying on biobased inputs for replacing massively produced single-use absorbents is limited by potential variations between production years, which could impair performance stability. Future work could implement tools such as digital twin models to aid the downstream development process, overcoming these upstream variations by simulating and optimizing processing steps for more consistent results.^50^ At the same time, although similar materials have demonstrated processability during upscaling in previous work,^49^ the recipes presented herein should be evaluated in pilot-scale extrusion, opening for further upscaling efforts.

### 3.5 Bioactivity Characteristics

Figure 6 shows the antioxidant activity of the developed foams measured in terms of radical scavenging activity (RSA) under 3 oxidative cycles. Assessing the antioxidant activity of these materials is important as antioxidants can neutralize odorous molecules, including –SH, NH_2_ and others, by chemical reactions with carbonyl and hydroxyl molecules.^51,52^ Notably, the use of Z and WG provided natural antioxidant properties to the developed foams, as observed in the control samples (Figure 6a). The result demonstrates the possibilities of using Z and WG for reducing the need for synthetic molecules to avoid the generation of smell in the samples. The RSA of the protein-based foams increases when Z is combined with SBC or through the mixture of WG, Z, and SBC. The RSA observed in the materials appears to be influenced by Z-SBC’s role in relation to WG-SBC. The differences between Z and WG under the incorporation of SBC can potentially be attributed to the amino acid composition of each protein, as well as changes in electrostatic balance and the exposition of available reactive groups caused by the alkaline conditions.^53,54^ On the other hand, this is not observed for WG incorporated with SBC as a blowing agent, where a drastic decline in RSA is observed. Plausibly, SBC may interfere with lowering the available reactive group exposition by cross-linking the protein under the prepared conditions, as previously shown by Bettelli et al.^49^ It has been shown that the cross-linking may affect the equilibrium between disulfide and sulfhydryl groups, which are responsible for the antioxidant activity in WG.^55^

**Figure 6:**
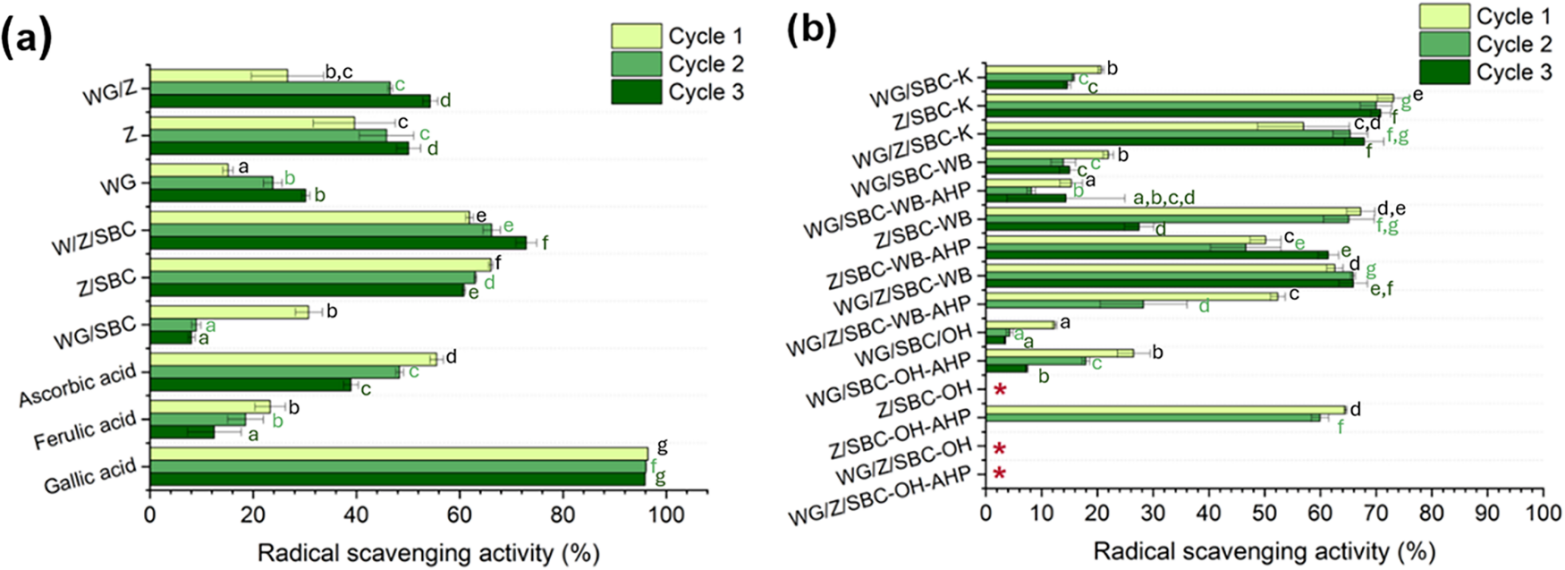
Antioxidant activity of (a) reference porous materials and common standards and (b) protein-based foams incorporated with Keratin (K), Wheat Bran (WB), and Oat Husk (OH) and delignified wheat bran (WB-AHP) and oat husk (OH-AHP). The red asterisk represents samples with no detected radical scavenging activity. Note: Different letters mean that the values are significantly different (*P* < 0.05).

Regarding the effect of biomass incorporation on the foams, none of the tested biomasses demonstrated an enhancing effect on the RSA of the foams. The incorporation of delignified OH (OH-AHP) and WG contributed to a weakening of the RSA (Figure 6b). This effect could be attributed to the higher alkaline pH resulting from the delignification process or to the disruption of protein network bonds, which are crucial for efficient electron transfer within the protein structure.^55^ Nevertheless, the incorporation of Keratin and WB without delignification into the WG/Z-SBC blends did not reduce the RSA compared to the reference. Although the long-term stability of the antioxidant effect requires further investigation, the materials maintained promising radical scavenging properties under a high oxidative environment and across multiple oxidative cycles. These findings support their potential as sustainable alternatives to synthetic antioxidants in sanitary applications.

Complementarily, the potential antimicrobial activity of the porous materials was investigated (Figure S16). In sanitary pads and hygiene products, antimicrobial activity is essential for protecting humans from pathogens and supporting the healthy growth of the natural vulvovaginal microbiota.^56^ The results revealed that most of the analyzed samples exhibited surface antimicrobial activity, as evidenced by the clear samples observed after contact with Gram-positive and negative bacterial strains. However, for samples WG/Z/SBC (Figure S161b) exposed to *E. coli*, and WG/Z/SBC-Keratin (Figure S162d) and WG/Z/SBC-WG (Figure S162e) exposed to *S. epidermidis*, microbial growth could be seen in the contact surface (Figure S161b′, 2d′, and 2e′, respectively) characterized for a completely opaque appearance. Z/SBC (Figure S161a,a′) exhibited microbial resistance across all evaluated strains, in contrast to the WG/Z/SBC blend (Figure S161b,b′), suggesting that WG may facilitate microbial growth in the blends. The various biofillers investigated did not enhance the antimicrobial properties of the protein-based foams. Thus, the antimicrobial properties of protein-based foams appear to be primarily governed by Z/SBC rather than other composite components, possibly due to electrostatic interactions and the exposition of reactive amino acid residues, which are also responsible for its antioxidant property.^55^

### 3.6 Thermomechanical Properties

#### 3.6.1 Materials’ Processing Window

The rheological properties of protein blends were analyzed to assess the impact of biofiller on processability. Figure 7a–c illustrates the temperature ramps of formulations containing Keratin, oat husk (OH), and wheat bran (WB), with and without sodium bicarbonate (SBC). WG-based formulations exhibited a gradual decrease in modulus with increasing temperature, consistent with previous studies (Figure 7a,a1).^57^ In contrast, Z-based blends exhibited an inflection point associated with the glass transition temperature (Tg), indicating a higher tendency to flow than WG, which affected the blend behavior (see Figure 7b,b1).

**Figure 7:**
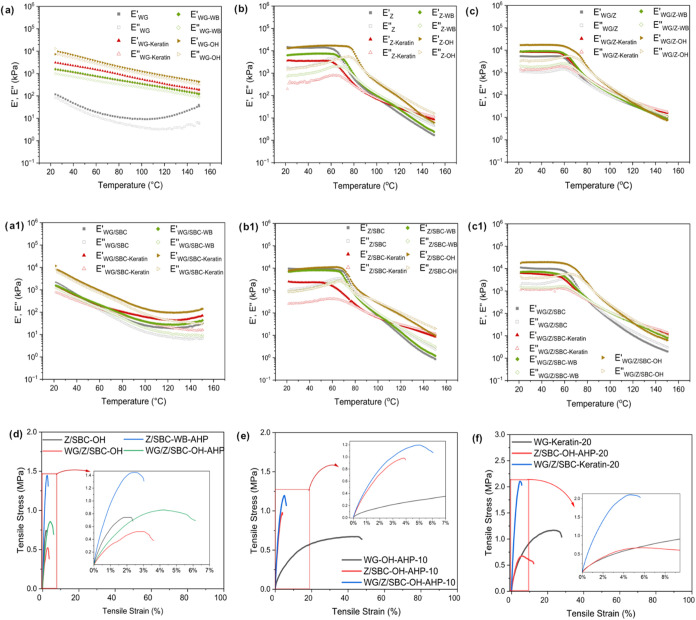
Temperature ramps of the different formulations with no biofiller, Keratin, wheat bran (WB) and oat husk (OH) for (a) WG, (a1) WG/SBC, (b) Z, (b1) Z/SBC, (c) WG/Z and (c1) WG/Z/SBC. Mechanical properties of the best swelling performance extruded formulations: tensile stress–strain curves of the extruded formulations (d) with as-received biofiller and with delignified biomass, (e) 10 wt % of biofiller, and (f) 20 wt % of biofiller.

The addition of OH increases the modulus values, resulting in stiffer and more viscous systems, ascribed to its larger particle size compared to WB and Keratin (Figure S14). SBC does not alter the thermal behavior of the formulations, except for the WG/SBC-based systems, which harden with temperature and correlate with the difficulties in extruding these formulations. Overall, all formulations are extrudable above 80 °C, a lower temperature than conventional polyolefins or PLA/PCL (≥180 °C). Here, postcooling is recommended to prevent cell wall collapse, as most blends exhibit significant modulus loss with increasing temperature. Among the biofillers tested, OH provides the highest modulus enhancement due to its larger structure.^58,59^

The rheological parameters of the formulations show that the samples with OH have the highest elastic modulus, complex viscosity and glass transition temperature (see Table S6). The formulations containing Keratin and WB only present a higher elastic modulus and complex viscosity than the reference ones when the recipe has WG without SBC (i.e., WG and WG/Z-based systems). The WB and OH formulations containing SBC exhibit higher critical strain values, suggesting that these samples can undergo significant deformation before reaching their failure point or undergoing property changes. This behavior is advantageous for postprocessing methods, allowing for the reshaping of the materials without compromising their properties. There are no differences between loss tangents of the different systems (comparing the systems based on the same formulation), being more solid at 20 °C those containing Z protein (lower loss tangent).

#### 3.6.2 Mechanical Properties of the Extruded Products

The tensile stress–strain curves for the formulations that exhibited the highest swelling performance are shown in Figure 7d–f. Among the different formulations, WG-based samples have ε_b_ above 40%, while Z-based samples exhibit greater stiffness with ε_b_ below 6% (Table 3). The Z sample containing 5 wt % of delignified WB (Z/SBC-WB-AHP) had the highest modulus, with *E* = 117 MPa (Figure 7d and Table 3). The delignification process could positively have altered the surface of the WB, leading to better matrix-biofiller interactions, and the particle size of WB (ca. 0.4 mm) could potentially function as a stabilizer in the cell walls, leading to a stiffer material (Figure S14). The effect of biofiller in the WG/Z blend shows that adding the as-received OH (5 wt %, WG/Z/SBC–OH) results in a modulus of 40 MPa, while delignifying the OH (WG/Z/SBC–OH-AHP-10) increased the modulus to 98 MPa (Table 3), which could be ascribed to changes in the matrix-biofiller interactions after altering the surface chemistry of the OH.

**Table 3:** Summary of the Tensile Properties of the Selected Extruded Formulations^a^

formulation	*E* (MPa)	σ_y_ (MPa)	σ_b_ (MPa)	ε_b_ (%)
Z/SBC–OH	75.17 ± 0.02^g^	0.7 ± 0.3^a,b^	0.69 ± 0.19^a,b^	2.3 ± 1.06^a^
WG/Z/SBC–OH	38.93 ± 0.01^f^	0.55 ± 0. 18^a^	0.51 ± 0.17^a^	3.48 ± 0.75^a,b^
Z/SBC-WB-AHP	117.62 ± 0.03	1.51 ± 0.2^d^	1.41 ± 0.22^d^	3.23 ± 0.84^a,b^
WG/Z/SBC–OH-AHP	97.51 ± 0.01^h^	0.87 ± 0.09^b^	0.83 ± 0.10^b^	4.78 ± 0.73^b^
WG–OH-AHP-10	17.65 ± 0.01^d^	0.68 ± 0.33^a,b^	0.60 ± 0.27^a,b^	54 ± 12.44^c^
Z/SBC–OH-AHP-10	2.56 ± 0.01^b^	1.03 ± 0.3^a,b,c^	0.99 ± 0.31^a,b,c,d^	4.09 ± 1.07^a,b^
WG/Z/SBC–OH-AHP-10	17.65 ± 0.01^e^	1.23 ± 0.27^b,c,d^	1.17 ± 0.29^b,d^	5.26 ± 1.09^b^
WG-Keratin-20	3.42 ± 0.01^c^	1.17 ± 0.14^c^	1.05 ± 0.10^c^	41 ± 2.09^c^
Z/SBC–OH-AHP-20	1.37 ± 0.01^a^	0.68 ± 0.18^a,b^	0.57 ± 0.14^a^	6 ± 1.94^b^
WG/Z/SBC-Keratin-20	17.75 ± 0.02^e^	2.16 ± 0.32^e^	1.9 ± 0.4^d^	5.27 ± 0.78^b^

a Note: Different letters mean that the values are significantly different (*P* < 0.05).

On the other hand, when the content of the biofiller increased to 10 wt %, Young’s modulus decreases, probably due to the disruption of the matrix continuity (Figure 7d and Table 3). When 20 wt % of Keratin fibers was added to WG (WG-Keratin-20), the extruded filaments showed an elongation at break of 41% (Table 3), which correlates with the uniform, continuous structure obtained for this formulation (see Figure 5d,d1). Furthermore, when the Keratin fibers are combined in the protein mixture (WG/Z/SBC-Keratin-20), the resulting modulus (*E* = 17.75 MPa) is comparable to that of the WG/Z/SBC–OH-10 sample. Hence, the results show that the nature, particle size, and the amount of the biofiller can tailor the absorbance properties but also the mechanical properties of the foams, leading to a step forward to more competitive materials.

It is worth remarking that the mechanical properties of the material can be related to the biofillers themselves (in their rawest form) and their size. Further work can assess these mechanical properties by standardizing the biofillers’ particle size and performing cross-comparisons, including the effect of this parameter on the delignification and extrusion performance. All in all, the results presented here demonstrate a promising pathway toward fully biobased foams that could potentially compete with commercial absorbents, also in terms of their mechanical properties. In previous work, pure gluten-based foam outperformed a commercial PUR foam in extensibility and achieved nearly 100% recovery after compression for up to 3 h.^13^ Building on these findings, the next step for protein blend matrices reinforced with biofillers would be to assess the tensile resilience, softness, and hysteresis properties, which are relevant for constructing a full prototype absorbent item.

## 4 Conclusions

The liquid absorbance and retention properties of extruded protein-based porous materials can be tailored and enhanced by simply upcycling oat husk, wheat bran, and Keratin fibers from industrial biomass waste. The liquid, mechanical, and bioactive properties of these materials offer a sustainable alternative to single-use plastic absorbents used in widely consumed products, such as sanitary pads. The developed foams exhibited a low density of 574 kg/m^3^ and an average pore size of 232 μm while maintaining mechanical properties suitable for medium-density applications. Among the untreated biofillers, oat husk showed the highest absorbance and retention in saline, increasing absorption by 66% with just 5 wt %. Delignification of oat husk and wheat bran further improved absorption, reaching 5 g/g compared to 4 g/g with untreated biofillers. The addition of 20 wt % Keratin further enhanced liquid absorption, achieving 6.5 g/g, approximately 40% of the capacity of synthetic foams used in sanitary pads. Zein-based foams also contributed to bioactivity, providing antioxidant and potential surface antimicrobial properties. The findings highlight an alternative approach to producing extruded, porous absorbents from upcycled food waste biopolymers, utilizing energy-efficient processing temperatures and enhancing absorbance due to a more porous and interconnected network. This innovation supports the replacement of single-use plastics in sanitary applications and aligns with Single-Use Plastic (SUP) directives that ban disposable plastics.

## Supplementary Material



## Data Availability

Data will be made available on request.

## Notes

Additional experimental details, methods, results, including the illustration of the experimental setup; illustration of the formulation for the rheology measurements (Figure S1); experimental illustration for the delignification of the lignocellulosic biomasses, wheat bran and oat husk (Figure S2); FT-IR of the solutions during the delignification process (Figure S3); SEM images of the each step during the delignification process of wheat bran and oat husk (Figure S4); FT-IR, TGA, and SEM images of Keratin fibers (Figure S5); visual absorbance test of the as all components and glycerol loss of the extruded formulations (Figure S6); FT-IR, TGA, and SEM images of the as-received proteins, wheat gluten and zein (Figure S7); forces applied during extrusion (Table S1); SEM images of the extruded formulations at 5 wt % of biofiller content (Figure S8); physical characteristics of the extruded formulations at 5 wt % of as-received, delignified oat husk (OH-AHP) and wheat bran (WB-AHP), at 10 and 20 wt % of Keratin and delignified oat husk (OH-AHP) (Tables S2–S5); free swelling capacity in saline solution and sheep blood and centrifuged retention capacity in saline solution of the extruded formulations at 5 wt % of biofiller content (Figure S9); SEM images of the extruded formulations at 5 wt % delignified wheat bran and oat husk with the lowest swelling performance (Figure S10); SEM images of the extruded formulations at 10 and 20 wt % delignified oat husk and Keratin with the lowest swelling performance (Figures S11 and S12); pore size distribution of the samples with the highest performance in FSC in saline (Figure S13) and particle size distribution of biofillers, oat husk (OH), wheat bran (WB), and Keratin fibers (Figure S14); increase of swelling in saline solution and blood between 2 cycles of swelling of PUR and WG/Z/SBC-Keratin-20 (Figure S15); antimicrobial activity images of the extruded samples (Figure S16) (PDF)
