# Supplementary material for: Extruded Porous Protein–Lignocellulosic Blends as Fully Bio-Based Alternative to Single-Use Absorbent Plastics
Source: ACS Appl Polym Mater. 2025 Sep 23;7(19):13099–113. doi: 10.1021/acsapm.5c02445 (PMC12519569; doi:10.1021/acsapm.5c02445)
Supplement: Supplementary file 1 [file ap5c02445_si_001.pdf]

Supplementary Information

Extruded Porous Protein–Lignocellulosic Blends as Fully Bio-Based Alternative to Single-Use Absorbent Plastics

Athanasios Latras[1]*, Pamela F. M. Pereira[2], Amparo Jiménez-Quero[2], Karin Odelius[1], Mercedes Jiménez-Rosado[3], Antonio J. Capezza[1]*

1. Department of Fibre and Polymer Technology, KTH Royal Institute of Technology, Stockholm, SE-100 44, Sweden
2. Division of Industrial Biotechnology, Department of LIFE Sciences, Chalmers University of Technology, Gothenburg, SE-412 96, Sweden
3. Department of Applied Chemistry and Physics, University of Leon, Leon, ES-24009, Spain

*Correspondence: latras@kth.se / ajcv@kth.se, Tel: +46 76230165

Biofillers characterization

Lignin content

The lignin content of the biofillers was determined following the technical report by Sluiter et al[1]. In summary, lignocellulosic biofillers were subjected to sulfuric acid hydrolysis, followed by the gravimetric quantification of acid-insoluble lignin (Klasson lignin). The soluble lignin content was measured from the hydrolyzed fraction using an absorbance at 205 nm, according to the recommendations from the ISO standard No. 21436:2020[2].

Monosaccharides composition

The monosaccharide composition of the biofillers was determined by trifluoroacetic acid (TFA) hydrolysis, as described by Albersheim et al[3]. Determinations were performed using high-performance anion-exchange chromatography coupled to pulsed amperometric detection (HPAEC-PAD) on a Dionex ICS-6000 system (Thermo Fisher, USA). A column CarboPacTM PA20 (3 x 150 mm) was used for separation at 0.4 $mL.min^{-1}$ and the gradient was performed according to described in Massironi et al[4]. Quantification was carried out against a calibration curve built with neutral sugars (fucose, arabinose, rhamnose, galactose, glucose, xylose, and mannose) along with uronic acids (galacturonic and glucuronic acids).

Starch content

The starch content was enzymatically determined using the Total Starch kit from Megazyme (Wicklow, Ireland), and the measurements were performed at 510 nm with glucose as the standard.

Phenolic acids analysis

Phenolic compounds in the biofillers were complementarily determined by ultra-high-performance liquid chromatography (UHPLC) using a Vanquish HPLC-PDA system (Thermo Scientific, MA, USA) with a C18 column (Luna 5u Phenomenex 100 A, 4.60 x 160 mm). Samples were injected using 25 µL, eluted at a flow rate of 0.7 mL/min and recorded at 280 nm. The gradient consisted of 0.1% formic acid and methanol as eluent A and B, respectively, as follows: 0-50 min, 95 % A and 5% B; 50-65 min, 50 % A and 50% B; 65-80 min, 95 % A and 5% B. Phenolic compounds quantification was performed against standards prepared at concentrations ranging from 0.005 to

0.1 g.L^{-1} including hydroxybenzoic acids (gallic acid, gentisic acid, vanillic acid, and chlorogenic acid), hydroxycinnamic acids (caffeic acid, ferulic acid, sinapic acid, p-coumaric, cinnamic acid) and flavonoids (catechin, rutin, and quercetin) standards. Prior to analysis, samples were saponified with 2 M NaOH at 30 °C and stirred overnight, followed by ethyl acetate partition (1:2, v/v), which was repeated three times.

Reswelling test

To evaluate the reswelling capacity of the extruded WG/Z/SBC-Keratin-20 and a commercial PUR foam (extracted from a sanitary pad), approximately 200 mg of each material was weighed and immersed in saline solution and defibrinated sheep blood for 30 min. After swelling, the samples were left to dry in the fume hood for 30 min. Additionally, one sample was dried overnight in an oven at 45 °C after the first swelling. Following the drying step, all samples were subjected to a second 30 min swelling in saline solution and blood, respectively. The difference between the two swelling cycles was calculated and reported as a percentage (%) average of triplicates.

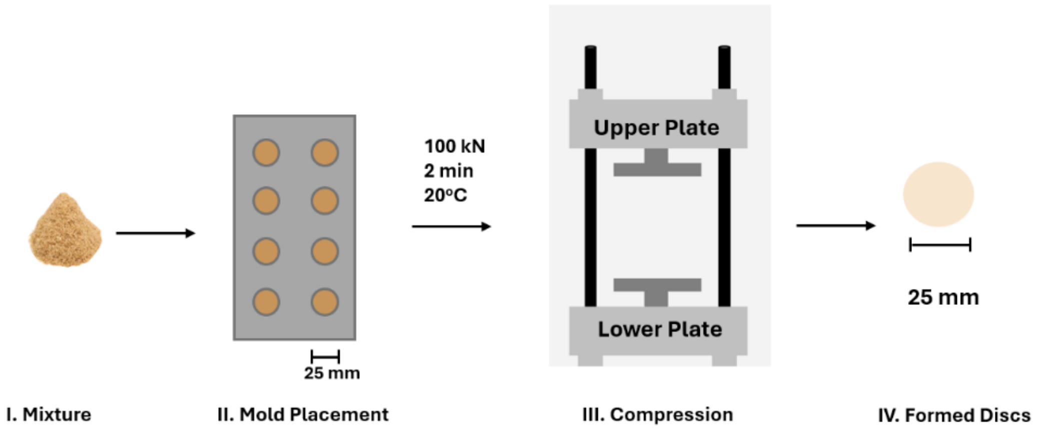

Figure S1. Schematic illustration of the manufacturing of protein blend discs for rheology measurements.

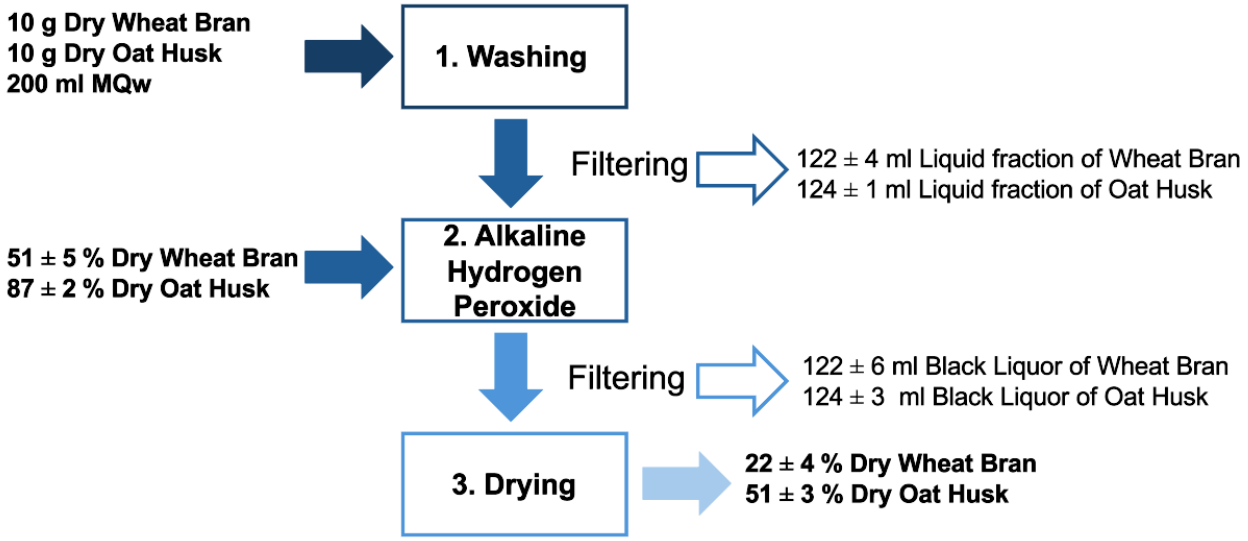

Figure S2. Illustration of the delignification process of the biofillers, wheat bran and oat husk, and their yields.

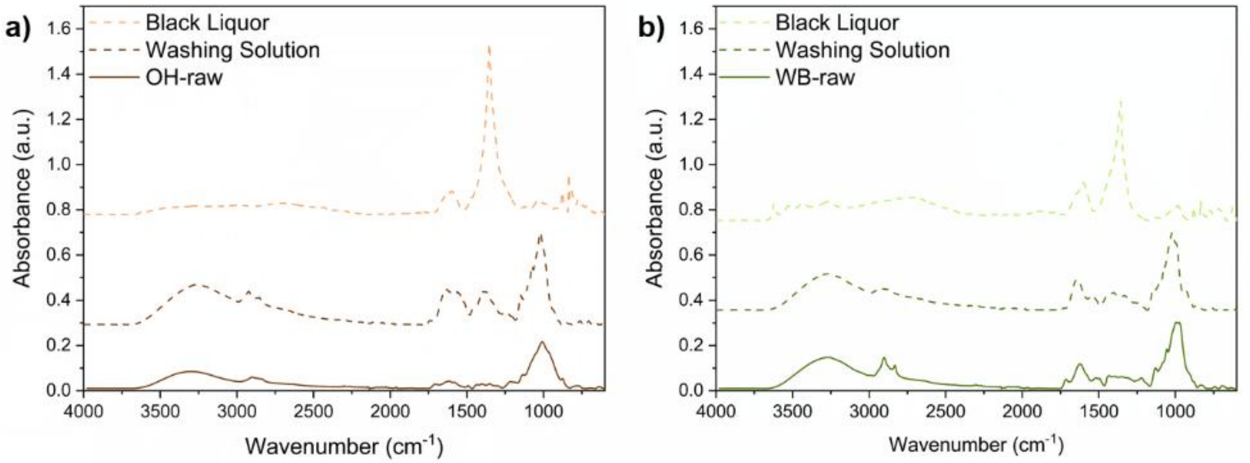

Figure S3. FT-IR profiles of the (a) OH and (b) WB raw and the solutions after washing and after delignification, respectively.

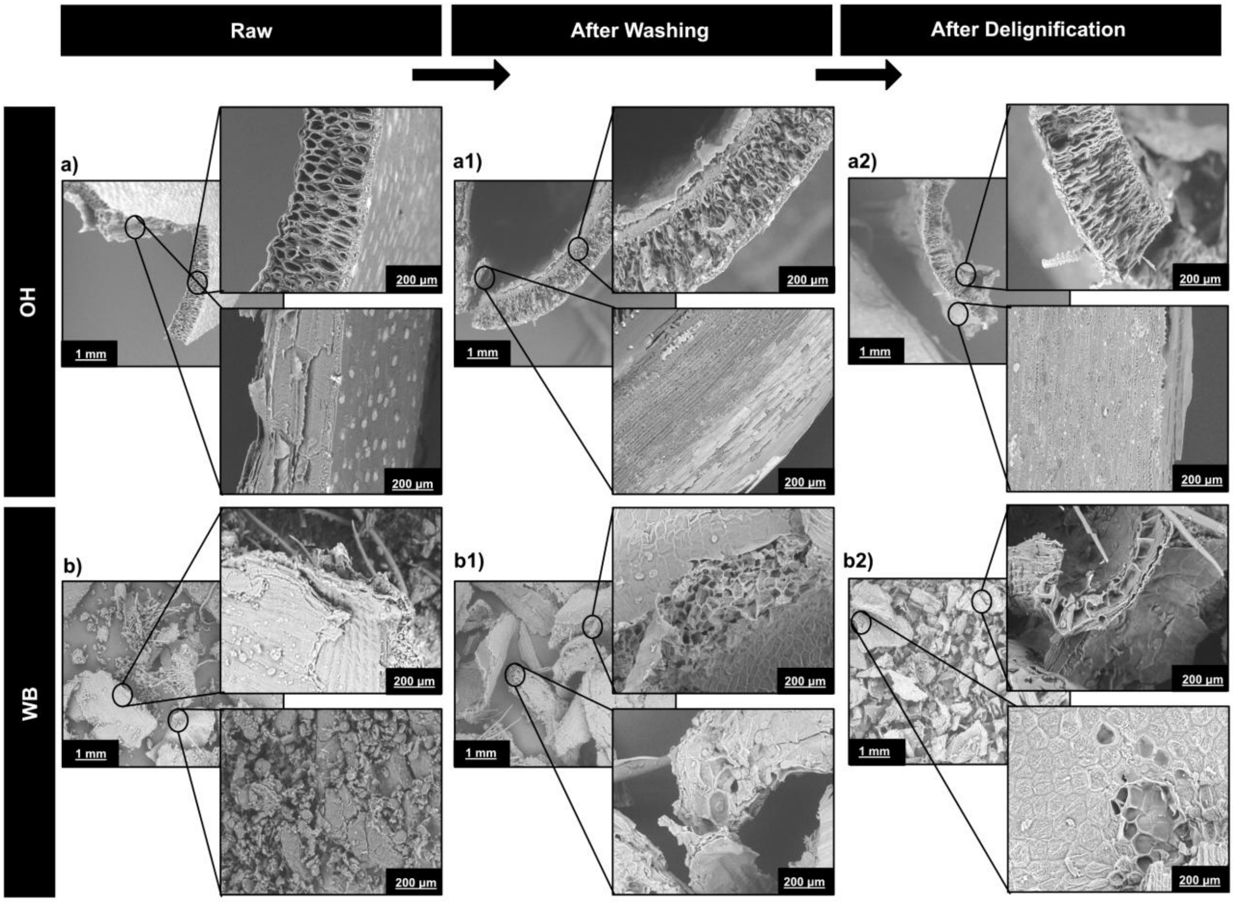

Figure S4. Microstructure of raw (a) oat husk and (b) wheat bran, (a1, b1) after washing, and (a2, b2) after delignification, respectively.

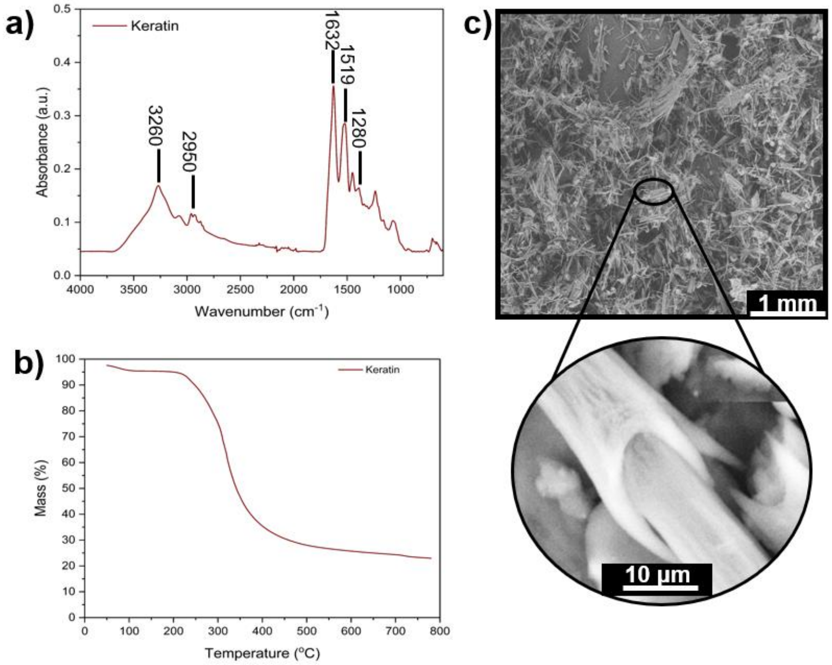

Figure S5. (a) FT-IR profile, (b) TGA profile, and (c) microstructure of Keratin fibers.

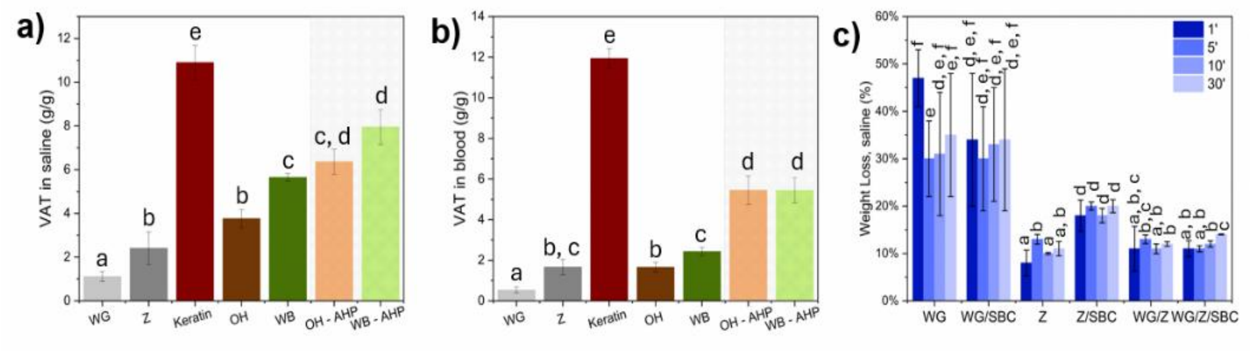

Figure S6. Visual Absorbance Test (VAT) (a) in saline (0.9 % wt. of NaCl) and (b) in the blood for the pure proteins (WG and Z), Keratin fibers, and as-received/delignified WB and OH, respectively, and (c) the glycerol weight loss during free swelling capacity (FSC) in saline from WG, Z, and WG with/without SBC. Note: Different letters mean that the values are significantly different ($P < 0.05$).

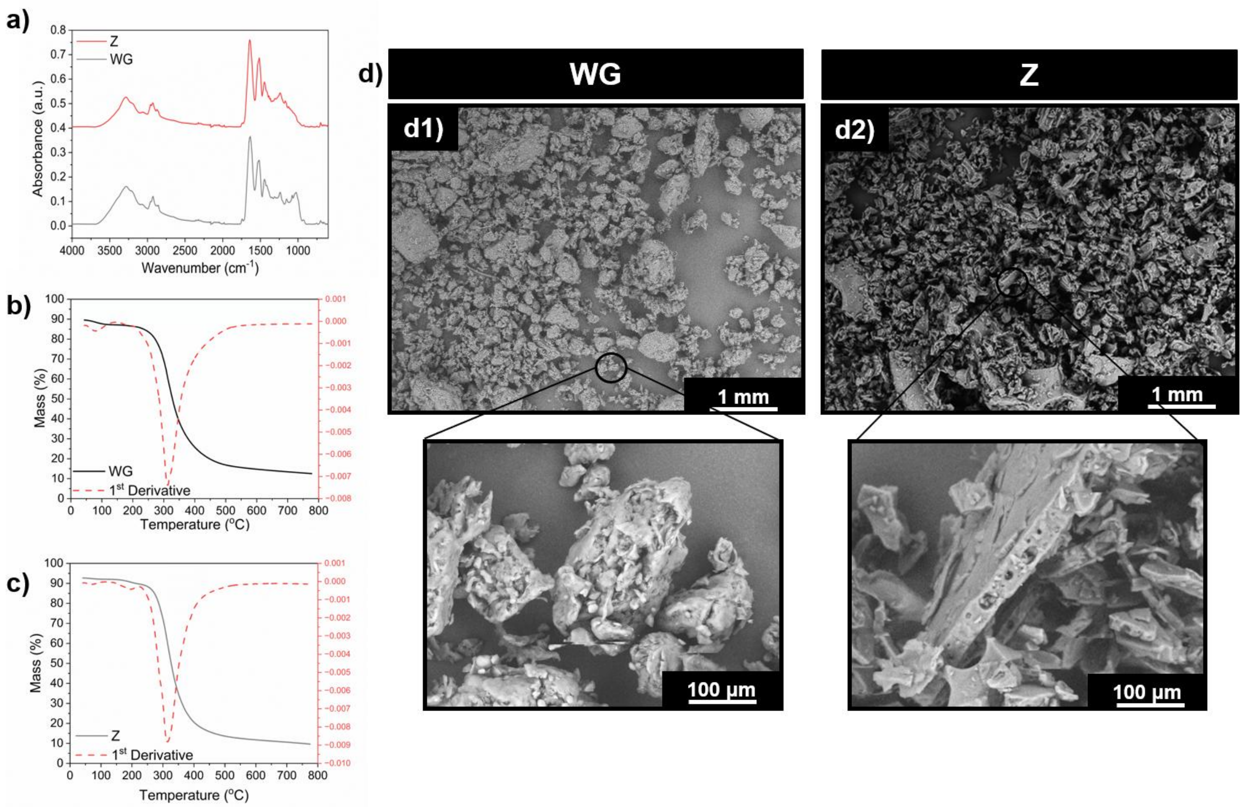

Figure S7. (a) FT-IR and (b-c) TGA and Derivative thermogravimetric profiles of the proteins (WG and Z) and (d) their microstructure.

Table S1. Forces applied during the extrusion of various formulations.

**Sample**	**Force (N)**						
	**No Biofillers**	**Keratin**	**WB**	**OH**	**OH-AHP**	**OH-AHP-10**	**OH-AHP-20**
WG	308 ± 155^{b}	287 ± 86^{d}	236 ± 29^{b}	416 ± 14^{d}	348 ± 90^{c}	412 ± 94^{c}	**NE**
WG/SBC	258 ± 84^{b}	223 ± 41^{d}	181 ± 54^{b}	418 ± 21^{d}	272 ± 7^{c}	**NE**	**NE**
Z	47 ± 10^{a}	43 ± 7^{a}	$109 \pm 24^{a,b}$	259 ± 10^{b}	89 ± 23^{a}	$259 \pm 140^{a,b}$	343 ± 253^{a}
Z/SBC	41 ± 10^{a}	39 ± 10^{a}	89 ± 9^{a}	$216 \pm 50^{a,b}$	71 ± 51^{a}	123 ± 29^{a}	394 ± 289^{a}
WG/Z	61 ± 26^{a}	106 ± 7^{c}	178 ± 63^{b}	333 ± 38^{c}	190 ± 17^{b}	239 ± 78^{b}	362 ± 21^{a}
WG/Z/SBC	42 ± 7^{a}	$82 \pm 26^{b,c}$	$97 \pm 21^{a,b}$	210 ± 12^{a}	161 ± 14^{b}	110 ± 31^{a}	372 ± 35^{a}

Note: NE means Not Extrudable. Different letters mean that the values are significantly different ($P < 0.05$).

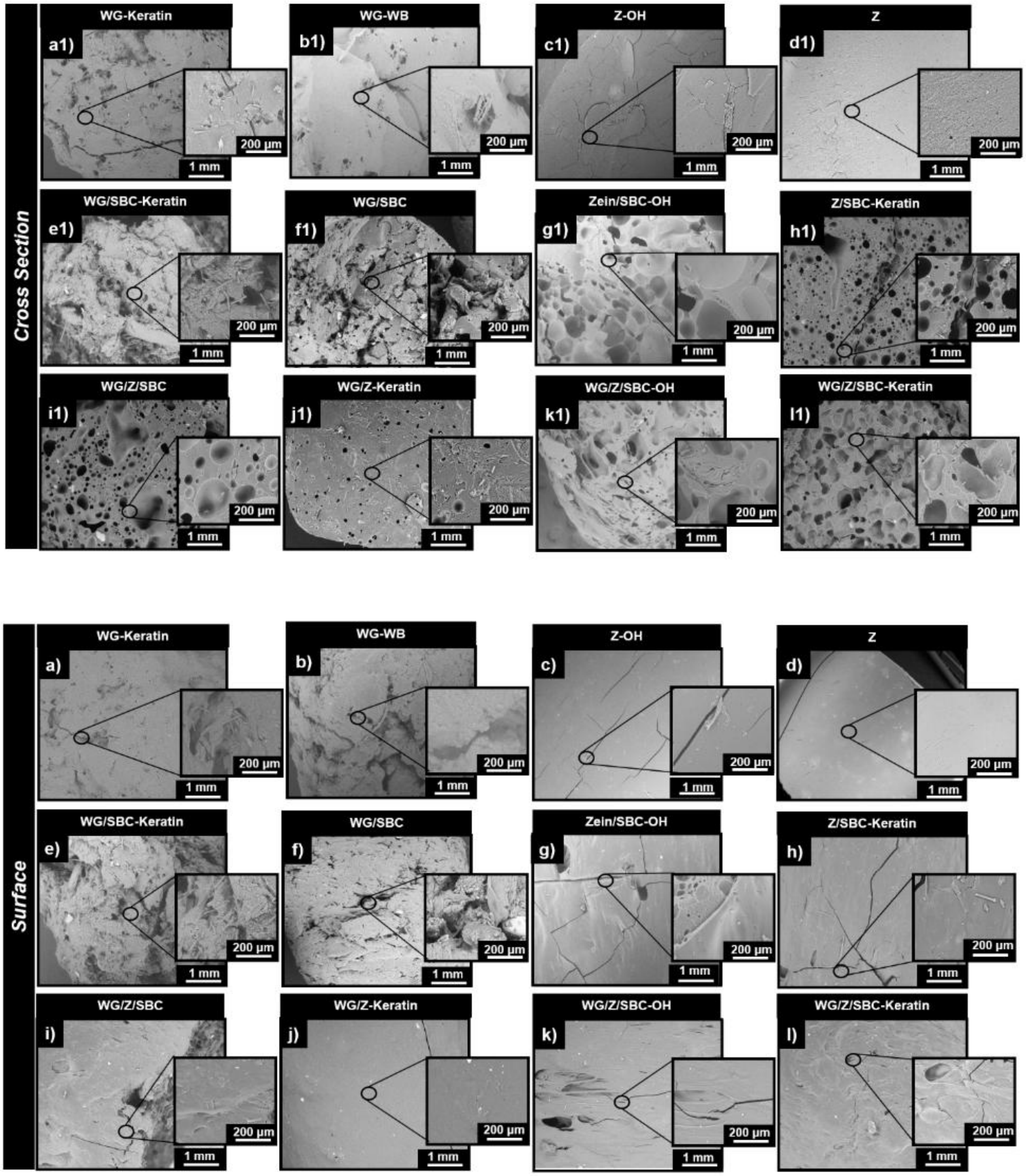

Figure S8. The microstructure of the (a1-l1) cross-section and (a-l) surface of the extruded materials with as-received biofiller. The samples represent those having the highest and lowest performance in the saline solution free swelling test.

Table S2. Physical characteristics of the samples with as-received biofiller 5 wt.%.

**Formulation**	**Expansion ratio (ER)**	**Apparent Density (kg/m^3)**	**Pore size (μm)**
WG	0.97 ± 0.06^{b}	$1146 \pm 166^{f, g, h}$	**NM**
WG/SBC	0.92 ± 0.12^{b}	$794 \pm 208^{c, e}$	**NM**
Z	1.25 ± 0.03^{e}	1351 ± 160^{h}	5 ± 22^{a}
Z/SBC	1.66 ± 0.08^{g}	807 ± 101^{e}	**NM**
WG/Z	1.40 ± 0.03^{f}	$1102 \pm 83^{f, g}$	$34 \pm 15^{a, b}$
WG/Z/SBC	1.92 ± 0.04^{h}	798 ± 80^{e}	**NM**
WG-Keratin	1.07 ± 0.02^{c}	919 ± 42^{e}	**NM**
WG/SBC-Keratin	0.96 ± 0.06^{b}	$735 \pm 123^{c, d}$	**NM**
Z-Keratin	$1.02 \pm 0.06^{b. c}$	$1045 \pm 90^{f, g}$	**NM**
Z/SBC-Keratin	$1.74 \pm 0.22^{g, h}$	610 ± 35^{c}	64 ± 36^{b}
WG/Z-Keratin	1.42 ± 0.05^{f}	1122 ± 51^{g}	$22 \pm 14^{a, b}$
WG/Z/SBC-Keratin	$1.52 \pm 0.08^{f, g}$	$656 \pm 43^{c, d}$	$35 \pm 44^{a, b}$
WG-WB	$0.99 \pm 0.07^{b, c}$	$1095 \pm 165^{f, g, h}$	**NM**
WG/SBC-WB	1.17 ± 0.04^{d}	680 ± 7^{d}	**NM**
Z-WB	1.49 ± 0.04^{f}	983 ± 52^{f}	**NM**
Z/SBC-WB	$1.81 \pm 0.35^{f, g, h, i}$	$672 \pm 126^{b, c, d, e}$	**NM**
WG/Z-WB	1.47 ± 0.09^{f}	$1018 \pm 40^{f, g}$	**NM**
WG/Z/SBC-WB	$1.83 \pm 0.18^{g, h}$	$646 \pm 143^{b, c, d, e}$	**NM**

WG-OH	0.64 ± 0.05^{a}	$1155 \pm 224^{f, g, h}$	**NM**
WG/SBC-OH	0.92 ± 0.05^{b}	465 ± 17^{a}	**NM**
Z-OH	$1.49 \pm 0.13^{f, g}$	$1132 \pm 100^{f, g, h}$	3 ± 13^{a}
Z/SBC-OH	2.51 ± 0.38^{i}	$587 \pm 88^{b, c, d}$	157 ± 85^{b}
WG/Z-OH	$1.46 \pm 0.02^{f, g}$	$1070 \pm 118^{f, g}$	**NM**
WG/Z/SBC-OH	1.95 ± 0.05^{h}	551 ± 12^{b}	120 ± 74^{b}

Note: Different letters mean that the values are significantly different (P < 0.05).

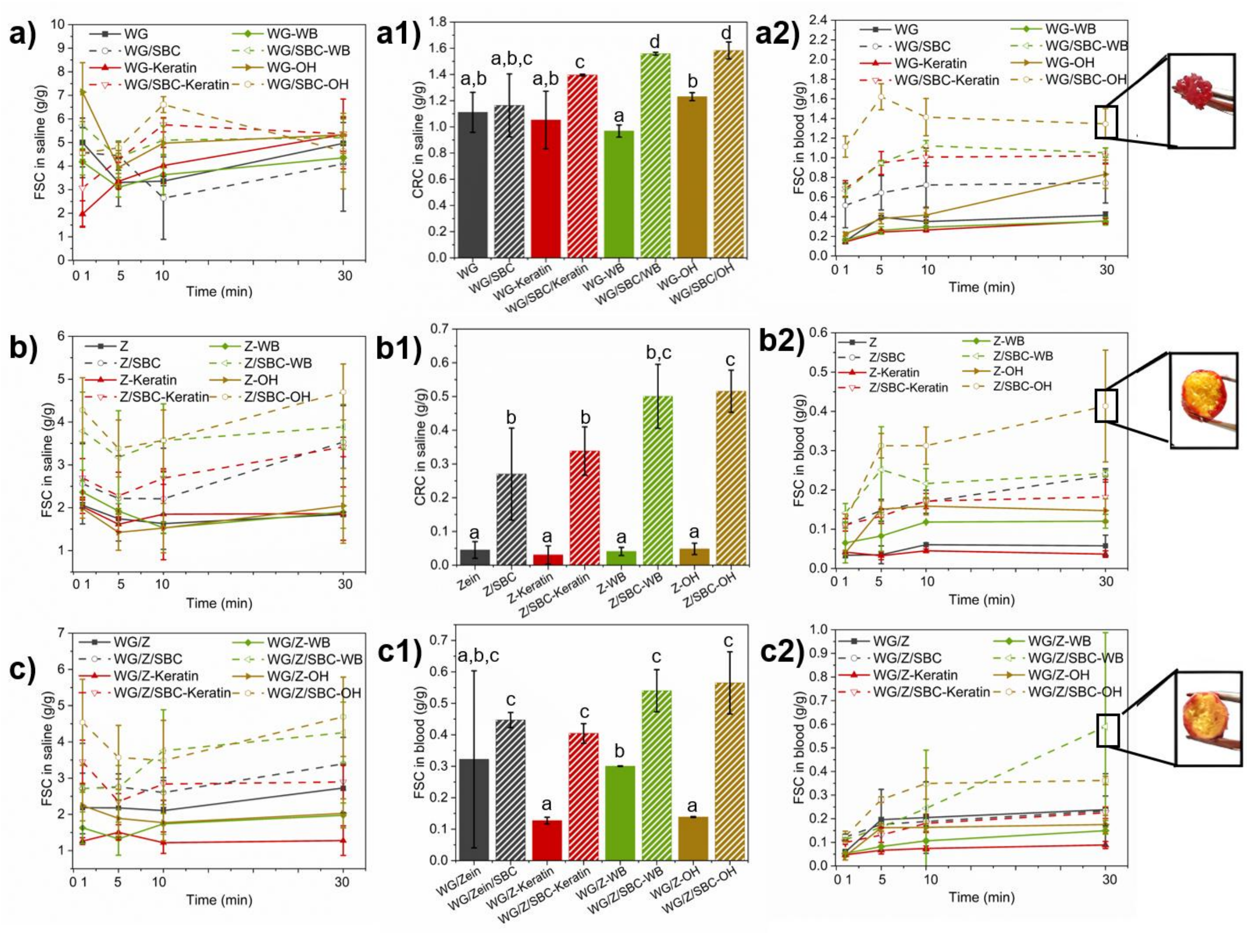

Figure S9. Free Swelling Capacity (FSC), Centrifuged Capacity (CRC) in saline solution and Free Swelling Capacity (FSC) in blood of (a-a1-a2) WG and WG/SBC, (b-b1-b2) Z and Z/SBC, and (c-c1-c2) WG/Z and WG/Z/SBC, with WB, OH, and Keratin, respectively. Note: Different letters in Fig. S9a1-c1 mean that the values are significantly different (P < 0.05).

Table S3. Physical characteristics of the samples with delignified biomass (wheat bran WB and OH oat husk).

Formulation	Expansion ratio (ER)	Apparent Density (kg/m^3)	Pore size (µm)
WG-WB-AHP	0.72 ± 0.04^{a}	1091 ± 114^{e}	**NM**
WG/SBC-WB-AHP	0.92 ± 0.12^{b}	$666 \pm 84^{a,b,c}$	**NM**
Z-WB-AHP	1.37 ± 0.03^{d}	1286 ± 30^{f}	**NM**
Z/SBC-WB-AHP	1.89 ± 0.03^{g}	699 ± 17^{c}	243 ± 107^{b}
WG/Z-WB-AHP	1.45 ± 0.02^{e}	$931 \pm 137^{d, e}$	**NM**
WG/Z/SBC-WB-AHP	1.59 ± 0.09^{f}	608 ± 11^{b}	**NM**
WG-OH-AHP	0.76 ± 0.02^{a}	$1046 \pm 91^{d, e}$	**NM**
WG/SBC-OH-AHP	$0.75 \pm 0.06^{a, b}$	844 ± 120^{d}	**NM**
Z-OH-AHP	1.25 ± 0.08^{c}	$979 \pm 72^{d, e}$	$277 \pm 515^{a, b}$
Z/SBC-OH-AHP	$1.55 \pm 0.08^{e, f}$	$701 \pm 53^{c, d}$	**NM**
WG/Z-OH-AHP	$1.15 \pm 0.11^{b, c}$	$1238 \pm 198^{e, f}$	41 ± 38^{a}
WG/Z/SBC-OH-AHP	1.57 ± 0.02^{f}	574 ± 13^{a}	232 ± 86^{b}

. Note: Different letters mean that the values are significantly different ($P < 0.05$).

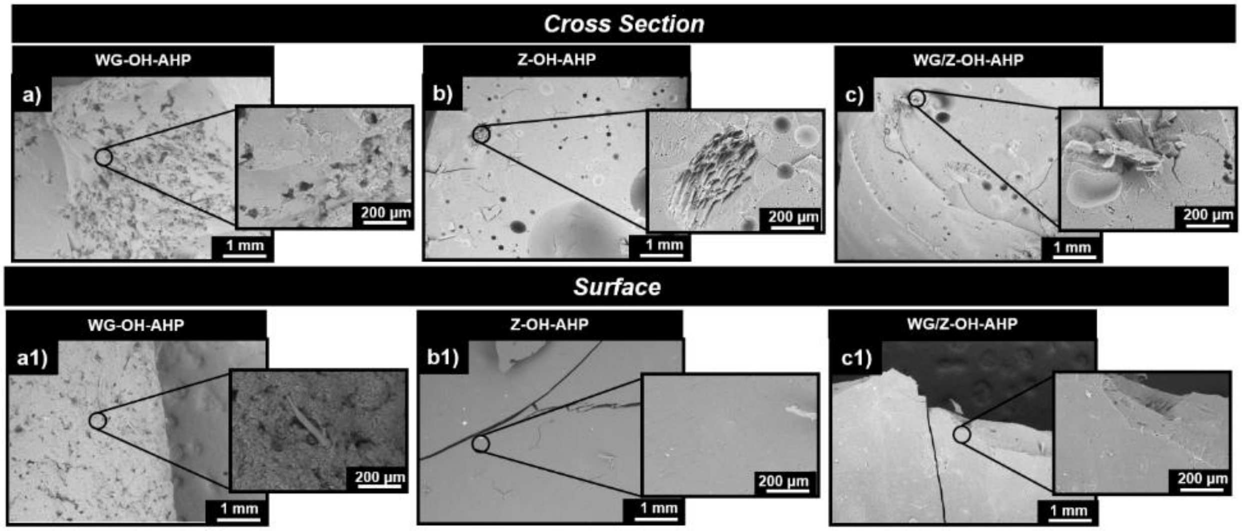

Figure S10. Microstructure of the (a-c) cross-section and (a1-c1) surface of the protein samples with the delignified biofiller without SBC. The samples shown are those having the lowest saline solution free swelling performance.

Table S4. Physical characteristics of the samples with 10 wt.% of delignified oat husk (OH-AHP) and Keratin.

Formulation	Content of Biofiller (g/100g protein)	Expansion ratio (ER)	Apparent Density (kg/m^3)	Pore size (µm)
WG-OH-AHP-10	10 %	0.71 ± 0.03^{a}	1453 ± 110^{c}	**NM**
WG/SBC-OH-AHP-10		**NE**		
Z-OH-AHP-10		1.1 ± 0.07^{c}	$1209 \pm 145^{b, c}$	**NM**
Z/SBC-OH-AHP-10		1.64 ± 0.15^{f}	649 ± 135^{a}	223 ± 106^{b}
WG/Z-OH-AHP-10		1.15 ± 0.08^{c}	$1339 \pm 200^{b, c}$	86 ± 60^{a}
WG/Z/SBC-OH-AHP-10		$1.25 \pm 0.18^{c, d, e,}$	$783 \pm 225^{a, b}$	120 ± 44^{a}
WG-Keratin-10		$0.76 \pm 0.02^{a, b}$	1513 ± 184^{c}	**NM**
WG/SBC-Keratin-10		0.82 ± 0.07^{b}	966 ± 176^{b}	**NM**
Z-Keratin-10		$1.38 \pm 0.02^{d, e}$	1059 ± 70^{b}	**NM**
Z/SBC-Keratin-10		1.76 ± 0.05^{f}	736 ± 34^{a}	**NM**
WG/Z-Keratin-10		1.35 ± 0.03^{d}	1008 ± 92^{b}	**NM**
WG/Z/SBC-Keratin-10		1.4 ± 0.02^{e}	$811 \pm 69^{a, b}$	**NM**

Note: Different letters mean that the values are significantly different ($P < 0.05$). NE means Not Extrudable.

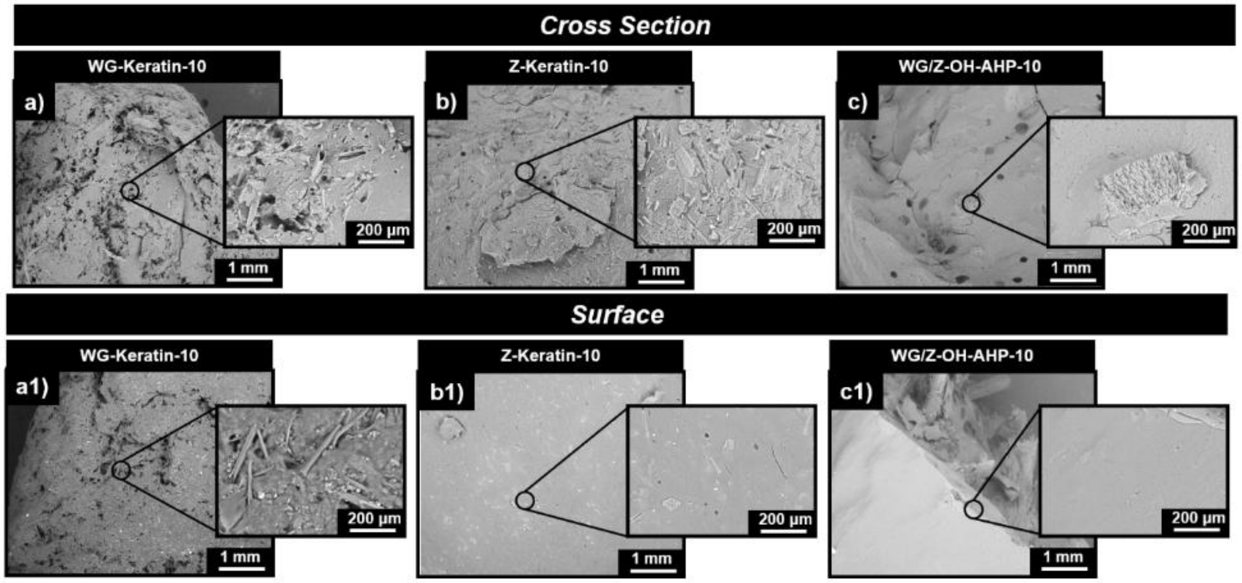

Figure S11. Microstructure of (a-c) cross-section and (a1-c1) surface of the protein samples with Keratin and 10 wt.% of delignified oat husk (OH-AHP) as biofiller and without SBC. The samples shown here represent those having the lowest free swelling performance.

Table S5. Physical characteristics of the samples of delignified oat husk (OH-AHP) and Keratin at 20 wt.% biofiller content.

Formulation	Content of Biofiller (g/100g protein)	Expansion ratio (ER)	Apparent Density (kg/m^3)	Pore size (µm)
WG-OH-AHP-20		NE		
WG/SBC-OH-AHP-20				
Z-OH-AHP-20		1.38 ± 0.02^{d}	852 ± 48^{b}	**NM**
Z/SBC-OH-AHP-20		1.53 ± 0.18^{d}	620 ± 177^{a}	213 ± 112^{b}
WG/Z-OH-AHP-20		0.79 ± 0.08^{a}	1364 ± 167^{e}	64 ± 34^{a}
WG/Z/SBC-OH-AHP-20	**20 %**	0.77 ± 0.04^{a}	$1294 \pm 187^{d, e}$	**NM**
WG-Keratin-20		0.76 ± 0.02^{a}	$1186 \pm 198^{c, d, e}$	**NM**
WG/SBC-Keratin-20		0.77 ± 0.03^{a}	$1149 \pm 233^{c, d, e}$	**NM**
Z-Keratin-20		1.29 ± 0.02^{c}	1044 ± 42^{c}	142 ± 14^{b}
Z/SBC-Keratin-20		1.65 ± 0.13^{e}	713 ± 92^{a}	**NM**
WG/Z-Keratin-20		1.09 ± 0.02^{b}	1134 ± 12^{d}	**NM**
WG/Z/SBC-Keratin-20		1.11 ± 0.02^{b}	$850 \pm 110^{a, b}$	114 ± 44^{b}

Note: Different letters mean that the values are significantly different ($P < 0.05$). NE means Not Extrudable.

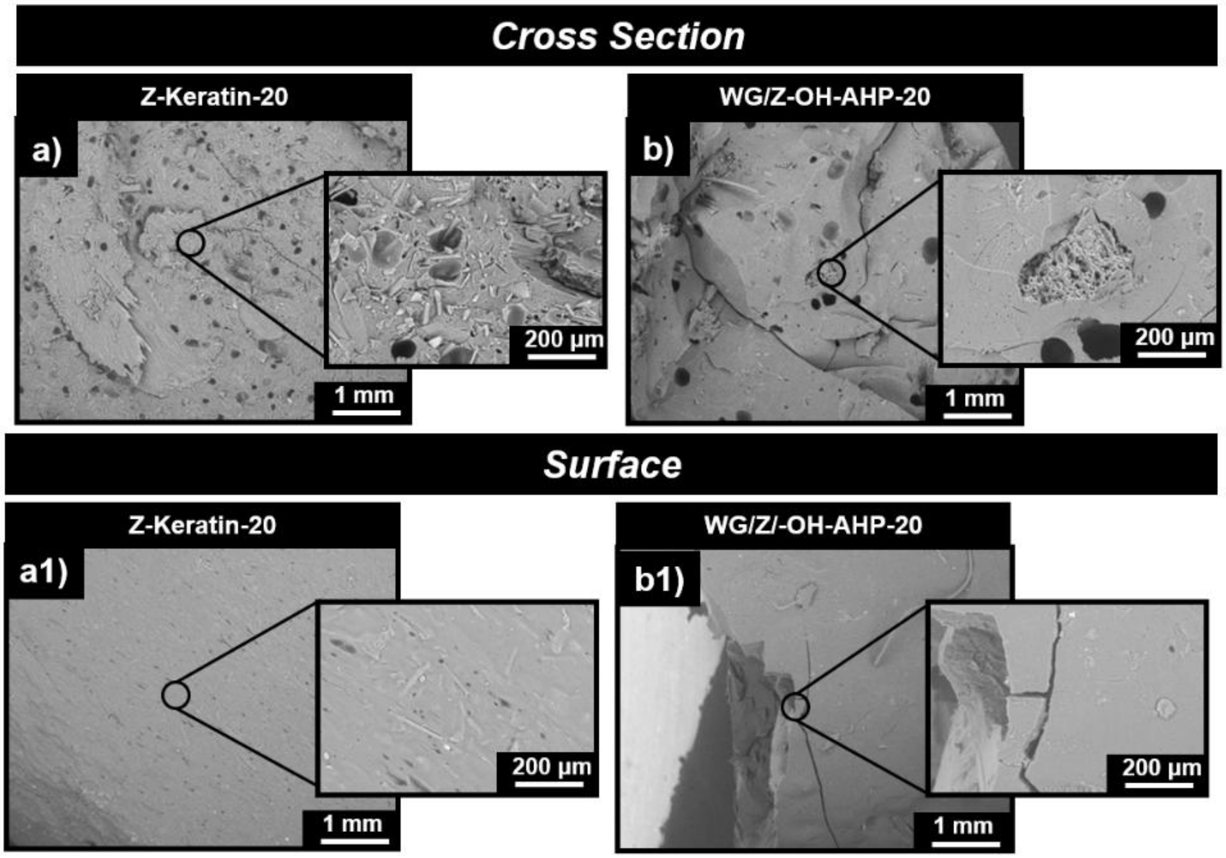

Figure S12. Microstructure of (a-b) cross-section and (a1-b1) surface of the protein formulations with Keratin and 20 wt.% of delignified oat husk (OH-AHP) as biofiller without SBC, with the lowest free swelling performance, respectively,

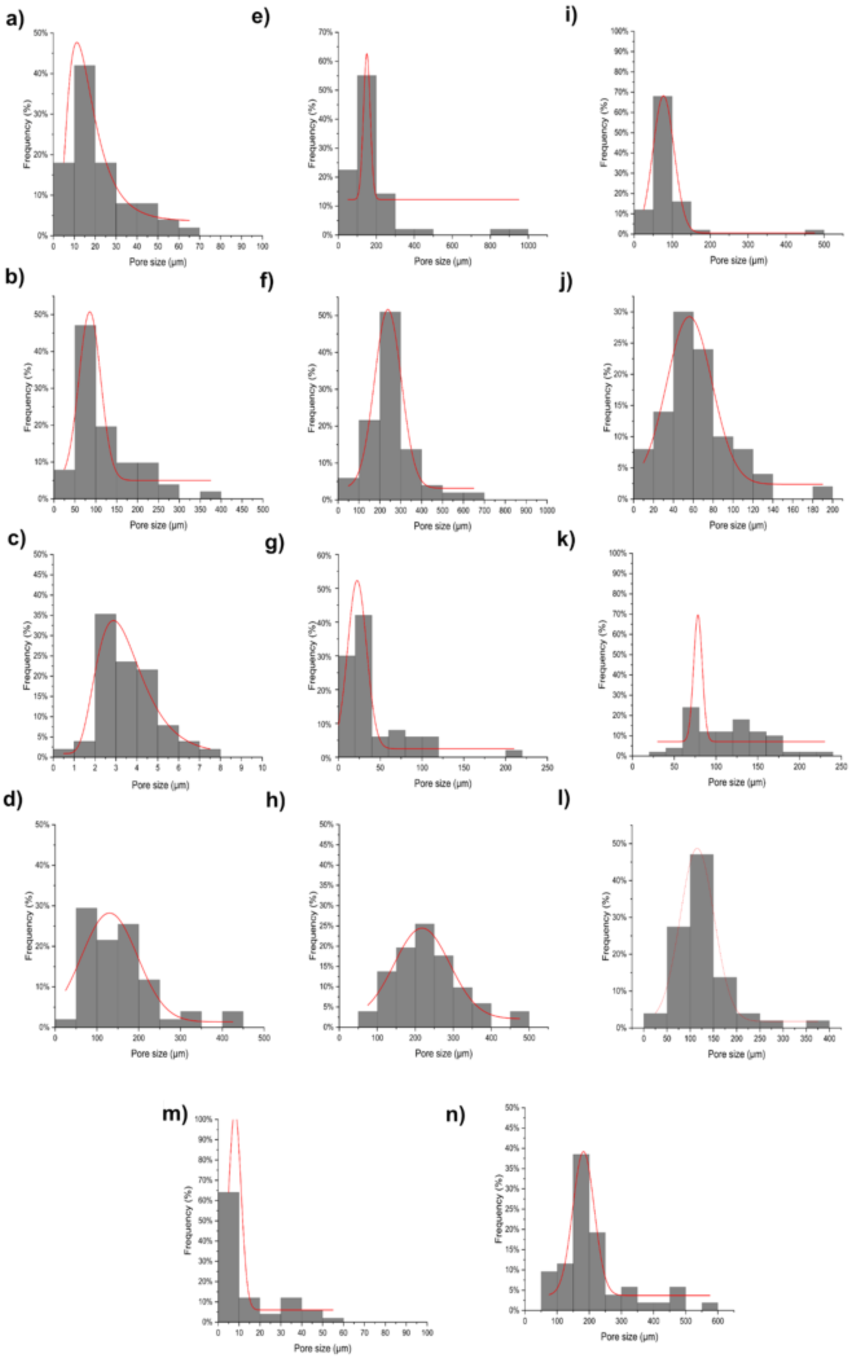

Figure S13. Pore size distribution in the cross-section microstructure of the best and least performing samples in FSC in saline: (a) WG/Z-Keratin, (b) WG/Z/SBC-OH, (c) Z-OH, (d) Z/SBC-OH, (e) Z-OH-AHP, (f) Z/SBC-WB-AHP, (g) WG/Z-OH-AHP, (h) WG/Z/SBC-OH-AHP, (i) WG/Z-OH-AHP-10, (j) WG/Z-OH-AHP-20, (k) WG/Z/SBC-Keratin-20, (l) WG/Z/SBC-OH-AHP-10, (m) Z-Keratin-20, and (n) Z/SBC-OH-AHP-20. Note: The WG-based samples are not measured because no pores are visually formed.

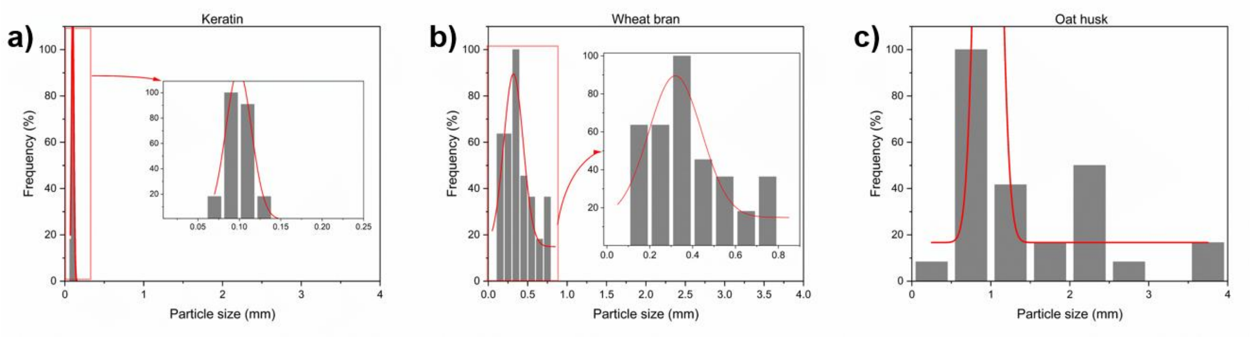

Figure S14. Particle size distribution of the biofillers (a) Keratin, (b) wheat bran (WB), and (c) oat husk (OH).

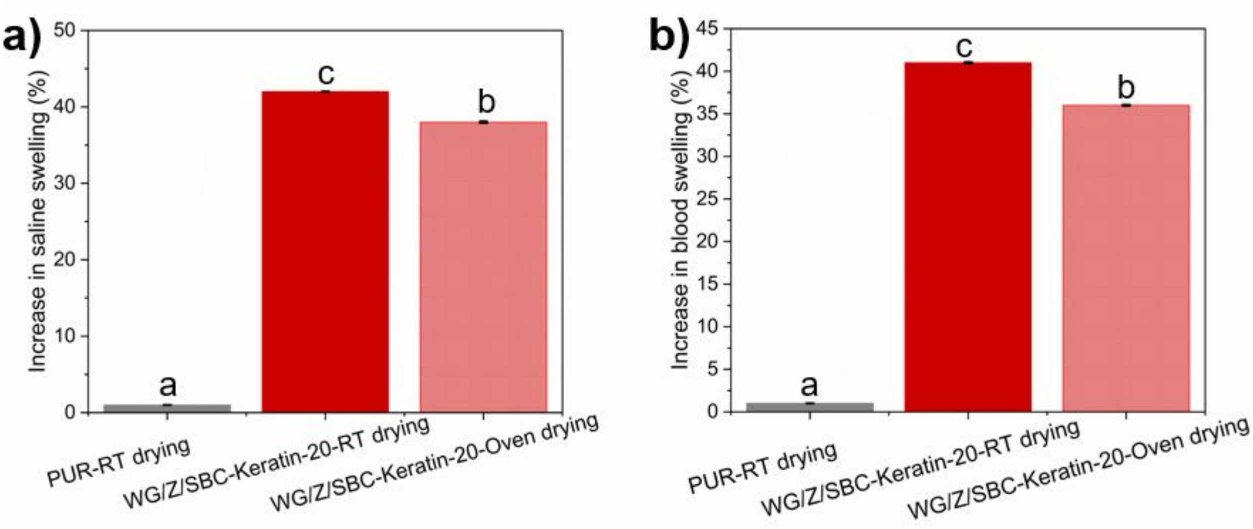

Figure S15. The percentage differences (a) in saline solution and (b) blood swelling between the 2 cycles of swelling (30 minutes) at room temperature of PUR and WG/Z/SBC-Keratin-20. The 30-minute swollen samples (PUR and WG/Z/SBC-Keratin-20) were left outside the liquids for 30 min and re-immersed in these for an additional 30 min (PUR-RT and WG/Z/SBC-Keratin-20-RT drying). The 30-minute swollen WG/Z/SBC-Keratin-20 were also dried overnight at 45 °C in an oven and re-immersed in the liquids for an additional 30 min (WG/Z/SBC-Keratin-20-Oven drying).

Table S6. Parameters obtained from the rheology measurements: critical strain (%), storage modulus (E_1') at 1.0 Hz, tan $(\delta)_1$ at 1.0 Hz, and complex viscosity η_1^* at 1.0 Hz, of the mixture formulations with and without SBC.

Formulation	Type of Biofiller		T_g (°C)	Critical Strain (%)	E'_1 (kPa)	tan $(\delta)_1$	η^*_1 (kPa.s)
WG	-	20°C	-	2.04 ± 0.52^{g}	253 ± 53^{a}	0.82 ± 0.03^{g}	52 ± 14^{a}
	Keratin		-	1.85 ± 0.66^{g}	855 ± 107^{c}	0.65 ± 0.01^{e}	163 ± 21^{c}
	WB		-	2.13 ± 0.05^{g}	466 ± 9.61^{b}	0.70 ± 0.02^{f}	90 ± 3^{b}
	OH		-	0.86 ± 0.03^{f}	2384 ± 511$^{d, e}$	0.85 ± 0.05^{g}	501 ± 120$^{d, e}$
WG/SBC	-	20°C	-	1.63 ± 0.50^{g}	475 ± 101^{b}	0.83 ± 0.02^{g}	99 ± 22^{b}
	Keratin		-	2.13 ± 0.03^{g}	368 ± 88^{a}	0.69 ± 0.01^{f}	71 ± 17^{a}
	WB		-	2.04 ± 0.01^{g}	476 ± 77^{b}	0.78 ± 0.04^{g}	97 ± 18^{b}
	OH		-	0.81 ± 0.01^{e}	2156 ± 363^{d}	0.83 ± 0.01^{g}	447 ± 120^{d}
Z	-	20°C	75 ± 4$^{a, b}$	0.07 ± 0.02^{a}	11075 ± 2528^{i}	0.19 ± 0.04$^{a, c, d}$	1799 ± 426^{i}
	Keratin		86 ± 4$^{c, d}$	0.31 ± 0.01^{d}	3648 ± 974^{e}	0.20 ± 0.01$^{b, c}$	593 ±160$^{d, e}$
	WB		82 ± 7$^{a, b, c, d}$	0.20 ± 0.13$^{a, b, d}$	6426 ± 379$^{g, h}$	0.17 ± 0.03$^{a, b, c}$	1039 ± 67$^{g, h}$
	OH		91 ± 3^{d}	0.18 ± 0.01$^{c, d}$	14144 ± 1036^{i}	0.18 ± 0.07$^{a, b, c, d}$	2290 ± 170$^{h, i}$
Z/SBC	-	20°C	79 ± 4$^{a, c}$	0.14 ± 0.06$^{a, b, c, d}$	8216 ± 3373$^{f, g, h, i}$	0.22 ± 0.01$^{c, d}$	1339 ± 547$^{g, h, i}$
	Keratin		85 ± 3$^{c, d}$	0.26 ± 0.07^{d}	2586 ± 237$^{d, e}$	0.21 ± 0.02$^{b, c, d}$	421 ± 37^{d}
	WB		79 ± 2^{b}	0.17 ± 0.03$^{c, d}$	6856 ± 1088$^{f, g, h}$	0.18 ± 0.01^{b}	1110 ± 179$^{f, g, h}$
	OH		91 ± 5^{d}	0.07 ± 0.01^{a}	10862 ± 3507$^{h, i}$	0.14 ± 0.1^{a}	1746 ± 564^{i}
WG/Z	-	20°C	84 ± 2^{c}	0.31 ± 0.07^{d}	4044 ± 529^{e}	0.24 ± 0.01^{d}	663 ± 86^{e}
	Keratin		92 ± 7$^{c, d}$	0.17 ± 0.01^{c}	5788 ± 32^{f}	0.23 ± 0.01$^{c, d}$	945 ± 2^{f}
	WB		78 ± 2$^{a, b}$	0.16 ± 0.01^{c}	7151 ± 433^{h}	0.22 ± 0.01$^{c, d}$	1166 ± 72^{h}
	OH		91 ± 6$^{c, d}$	0.18 ± 0.01$^{c, d}$	12539 ± 2486^{i}	0.27 ± 0.01^{e}	2068 ± 406^{i}
WG/Z/SBC	-	20°C	72 ± 4^{a}	0.13 ± 0.04$^{b, c}$	7468 ± 683^{h}	0.26 ± 0.03$^{d, e}$	1229 ± 120^{h}
	Keratin		88 ± 3$^{c, d}$	0.34 ± 0.24$^{b, c, d}$	4766 ± 1318$^{e, f, g}$	0.27 ± 0.04$^{d, e}$	786 ± 209$^{e, f, g}$
	WB		79 ± 2^{b}	0.197± 0.125$^{a, b, c, d}$	6184 ± 333^{g}	0.22 ± 0.03$^{b, c, d}$	1009 ± 62$^{f. g}$
	OH		96 ± 3^{d}	0.11 ± 0.01^{b}	16271 ± 658^{i}	0.21 ± 0.01^{c}	2654 ± 113^{j}

Note: Different letters mean that the values are significantly different (P < 0.05).

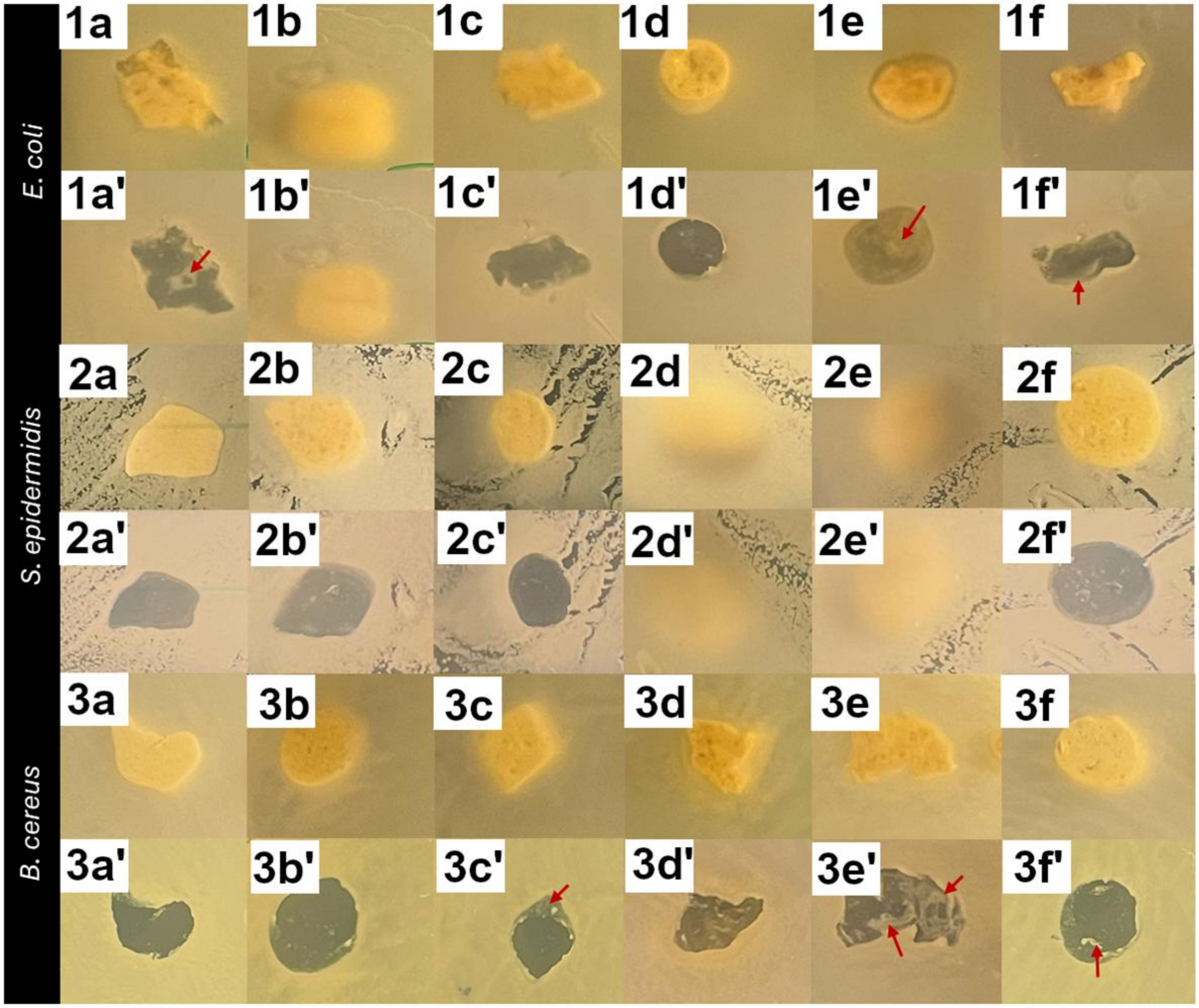

Fig S16. Antimicrobial activity from (a) Z/SBC, (b) WG/Z/SBC, (c) Z/SBC-Keratin, (d) WG/Z/SBC-Keratin, (e) WG/Z/SBC-WG, and (f) Z-SBC/WB materials and respective growth contact surface exposed to (1) *E. coli*, (2) *S. epidermidis,* and (3) *B. cereus*. NOTE: Red arrows mean bacteria growth on the surface

References

(1) Sluiter, A.; Hames, B.; Ruiz, R.; Scarlata, C.; Sluiter, J.; Templeton, D.; Crocker, D. Determination of Structural Carbohydrates and Lignin in Biomass: Laboratory Analytical Procedure (LAP) (Revised July 2011). **2008**.

(2) *ISO 21436:2020 - Pulps — Determination of lignin content — Acid hydrolysis method*.

(3) Albersheim, P.; Nevins, D. J.; English, P. D.; Karr, A. A Method for the Analysis of Sugars in Plant Cell-Wall Polysaccharides by Gas-Liquid Chromatography. *Carbohydr. Res.* **1967**, *5* (3), 340–345. https://doi.org/10.1016/S0008-6215(00)80510-8.

(4) Massironi, A.; Freire De Moura Pereira, P.; Verotta, L.; Jiménez-Quero, A.; Marzorati, S. Green Strategies for the Valorization of Industrial Medicinal Residues of Serenoa Repens Small (Saw Palmetto) as Source of Bioactive Compounds. *J. Environ. Manage.* **2024**, *370*, 122843. https://doi.org/10.1016/J.JENVMAN.2024.122843.
